# Phosphasalen Indium Complexes Showing High Rates and Isoselectivities in *rac*‐Lactide Polymerizations

**DOI:** 10.1002/anie.201701745

**Published:** 2017-04-05

**Authors:** Dominic Myers, Andrew J. P. White, Craig M. Forsyth, Mark Bown, Charlotte K. Williams

**Affiliations:** ^1^ Department of ChemistryImperial College LondonLondonSW7 2AZUK; ^2^ School of ChemistryMonash UniversityClaytonVIC3800Australia; ^3^ CSIRO ManufacturingBayview AvenueClaytonVIC3168Australia; ^4^ Department of ChemistryOxford University12 Mansfield RoadOxfordOX1 3TAUK

**Keywords:** indium, isoselectivity, polymerization, structure elucidation, synthetic methods

## Abstract

Polylactide (PLA) is the leading bioderived polymer produced commercially by the metal‐catalyzed ring‐opening polymerization of lactide. Control over tacticity to produce stereoblock PLA, from *rac*‐lactide improves thermal properties but is an outstanding challenge. Here, phosphasalen indium catalysts feature high rates (30±3 m ^−1^ min^−1^, THF, 298 K), high control, low loadings (0.2 mol %), and isoselectivity (*P*_i_=0.92, THF, 258 K). Furthermore, the phosphasalen indium catalysts do not require any chiral additives.

Polylactide (PLA) is the leading bioderived polymer and is a degradable replacement for petrochemical polymers in packaging, fibers, and biomedical devices.^1^ It is sourced from starch and is commercially synthesized by the metal‐catalyzed ring‐opening polymerization (ROP) of lactide (LA). Both the applications and material performances of PLA are improved by control of its stereochemistry. For example, atactic PLA is an amorphous polymer suitable as packaging for products having a short shelf life. In contrast, isotactic PLA is a semi‐crystalline polymer suitable for higher‐strength and longer‐lifetime applications.^2^ Moreover, co‐crystallization of the two enantiomeric homochiral PLA chains affords stereoblock/stereocomplex PLA, with a significantly higher melting temperature than isotactic PLA, a feature which facilitates processing and may enable applications as an engineering polymer.^3^ Stereoblock/complex PLA can be made from separate homochiral PLA chains.^4^ An alternative route involves *rac*‐lactide with an isoselective catalyst to yield, in one‐step, stereoblock PLA. This approach is intrinsically more attractive but is currently limited by the range and performance of the catalysts. Despite the research on PLA catalysis, there are remarkably few isoselective metal catalysts, with leading examples including chiral complexes of rare earth, group 13, and late‐transition metals.^5^ So far, chiral aluminium/salen complexes prepared from either binaphthyl or cyclohexyl diamines show the best isoselectivity.^6^ Nomura and co‐workers pioneered achiral aluminium/salen complexes, some of which also showed very high isoselectivity. The catalyst selectivity was highest using sterically hindered *ortho*‐phenolate substituents.^7^ The leading catalysts showed a probability of isotactic diad formation of *P*_i_>0.9 (343 K). The catalysts showed high polymerization control and, in some cases, showed narrow molecular weight distributions despite operating by a polymer exchange mechanism.6c Yet, the major limitation of the highest performing aluminium/salen catalysts are the low rates and requirement for high metal loadings (≥1 mol %), thus, more highly active isoselective catalysts are needed. One option is to replace aluminium with its heavier congener indium, a strategy shown to accelerate rates whilst maintaining control. Mehrkhodavandi and co‐workers have reported isoselective indium complexes, coordinated by chiral salen or phenoxide diamine ligands, with *P*_i_=0.77 (298 K).^8^

Considering the challenge of simultaneously improving the catalytic rate and isoselectivity, the selection of the ancillary ligand is important. So far, salen ligands have been extensively optimized at the diimine linkers, phenyl ring substituents, and even by changing the imine to an amine. We have recently demonstrated the potential of the phosphasalen ligand class, with yttrium and lutetium complexes affording *P*_i_ values ranging from 0.81 to 0.84 (298 K).5n–5p, 9 The phosphasalen ligand features iminophosphorane substituents which deliver greater electron density at the metal center compared to salen analogues, and thus accelerates rates. It is important to emphasize that the phosphasalen ligands do not contain any chiral diamines, that is, chiral at metal complexes can form and stereocontrol is also feasible by chain‐end control mechanisms. Further, the phosphorus atom substituents provide steric directing effects at the active site since directly analogous achiral salen yttrium complexes, which do not benefit from such substituents, are not stereoselective.6c, 10 Given the need for isoselective catalysts, the promising performance of indium salen catalysts was inspiring. Here, the first examples of phosphasalen indium catalysts, featuring ethylene diamine linkers, show high rates and isoselectivity (Figure 1).

**Figure 1:**
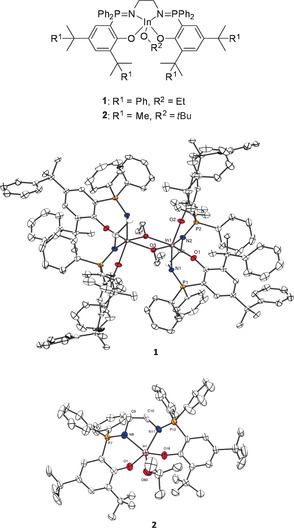
Molecular representation of structures of the initiators **1** (top left) and **2** (bottom right) obtained by X‐ray diffraction techniques, with hydrogen atoms and solvent molecules omitted for clarity (50 % probability ellipsoids).^21^ General schematic representation of the structures of **1** and **2** (top).

The most isoselective rare‐earth catalysts were coordinated by a pentadentate phosphasalen ligand featuring a triamine linker with an NH donor group. Initially, this same ligand was investigated for indium coordination chemistry but afforded a mixture of products, perhaps because of its higher denticity and/or the tendency of indium alkoxide complexes to dimerize. Using tetradentate phosphasalen ligands, indium complexes featuring ethylene linkers were isolated, and the *ortho* phenyl position was targeted for substitution as it is close to the active site. The ligands were synthesized using known or modified versions of the reported routes (see Scheme S1 in the Supporting Information).^12^ The synthesis of the indium complexes **1** and **2** was achieved in three steps and did not require any isolation of intermediates. Firstly, the phosphasalen ligands were deprotonated, by reaction with excess NaH, and the disodium salts were then reacted with InCl_3_. The indium chloride complexes were reacted with either KOEt or KO*t*Bu, and the solutions were filtered to remove the salt by‐products, thus allowing isolation of the pure indium alkoxide complexes **1** and **2** in 31 and 44 % yields, respectively (Figure 1; see Figures S1–S10). The reactions were all monitored using in situ ^31^P{^1^H} NMR spectroscopy, and at each stage, products showed characteristic singlets at different chemical shifts. It is relevant to note that although the complexes feature different alkoxide co‐ligands, these are the initiating groups in catalysis and so do not influence either rates of propagation or isoselectivity.

Both **1** and **2** showed singlets in the ^31^P{^1^H} NMR spectra, at *δ*=39.6 and 40.3 ppm, respectively, thus indicating the phosphorus atoms are in identical environments. The ^1^H NMR spectra showed the expected number and integration of signals for the phosphasalen ligands as well as for the alkoxides. Indium coordination was confirmed by the appearance of diastereotopic methylene environments for the backbone protons. In terms of the *ortho* phenyl substituents, **2** showed two distinct sharp singlets for both the methyl resonances, which were assigned to the *ortho*‐ and *para*‐phenyl groups.

In contrast, **1** showed two different methyl resonances for the *ortho*‐cumyl groups and both were asymmetrically broadened, which was indicative of restricted rotation. ^1^H ROESY NMR spectra showed exchange processes between the ethylene backbone signals and aromatic phenyl groups (see Figure S11). These likely involve free rotation of the phenyl groups and twisting of the ethylene backbone, and is consistent with the ^1^H NMR data. Additional crosspeaks were observed between the asymmetric methyl resonances of the *ortho*‐cumyl groups. VT‐NMR studies revealed that the *ortho*‐cumyl resonances undergo signal coalescence at high temperature (*T*≥318 K). A similar coalescence was also observed for the ethylene backbone signals, changing from two broad multiplets into a sharp doublet at 363 K (see Figure S12–S15).

The solid‐state structures were obtained by single‐crystal X‐ray diffraction (Figure 1). The compound **1** is an ethoxide‐bridged dimer with each indium atom exhibiting octahedral coordination geometry (see Figure S16 and Table S1). In contrast, the solid‐state structure of **2** is mononuclear and the indium is pentacoordinate with a geometry intermediate between trigonal bipyramid and square‐based pyramidal (*τ*=0.33; see Figures S17 and S18, and Table S2). The distinct nuclearities appear to be dominated by the nature of the alkoxide, with the smaller ethoxide co‐ligand (**1**) permitting a bridging coordination geometry. Perhaps more unexpected is the isolation of mononuclear **2**, given the strong precedence for dimeric [(κ^4^‐ligand)In(OR)]_2_ complexes in the literature.^13^ In polymerization catalysis there is an excess of monomer, which is a potential donor group, and THF (tetrahydrofuran) is the solvent, thus establishing the nuclearity of the complexes under related conditions is important. The complex **1** was analyzed by diffusion‐ordered NMR spectroscopy (DOSY) in both noncoordinating ([D_6_]benzene and [D_8_]toluene) and coordinating solvents ([D_8_]THF), and the diffusion coefficients and hydrodynamic radii (*r*_H_) were compared.

Whilst such analysis is only semi‐quantitative, the *r*_H_ values in THF are approximately half those obtained in benzene/toluene (*r*_H_ ${}^{[D{}_{8}]\mathrm{THF}}$ =4.7 Å, *r*_H_ ${}^{[D{}_{6}]\mathrm{benzene}}$ =6.8 Å, *r*_H_ ${}^{[D{}_{8}]\mathrm{toluene}}$ =7.0 Å, *r*_H_^solid‐state dimer^=8.1 Å; see Figures S19–S21). The complex **2** showed a mononuclear solid‐state structure and there was close agreement with hydrodynamic values in THF, and is thus strongly indicative of it adopting a mononuclear solution structure (*r*_H_ ${}^{[D{}_{8}]\mathrm{THF}}$ =6.2 Å, *r*_H_^solid‐state^=5.9 Å; see Figure S22). Therefore, it is reasonable to expect that both initiators are mononuclear under the polymerization conditions.

Both **1** and **2** were highly active for the ROP of *rac*‐LA and showed good polymerization control (Table 1; see Figures S23–S43). The typical polymerization conditions were in THF solutions, at 298 K, and used [LA]_0_=1 m and [catalyst]_0_=2 mm. By using **1**, polymerization proceeded rapidly, reaching near‐quantitative conversion in 1 hour (Table 1, entry 1). Whilst still rapid in the broader context of this catalysis,^14^ using **2**, under identical reaction conditions resulted in reactions that were more than four times slower. Linear semi‐logarithmic plots for the ROP of *rac*‐LA by **1** and **2** confirmed that the rates were first‐order with respect to monomer concentration (Figure 2; see Figures S44–S46). The rate coefficients were determined as *k*_obs_=8.27±0.48×10^−4^ s^−1^ and 1.94±0.04×10^−4^ s^−1^, respectively. Polymerizations for **1** and **2** also showed first‐order rate dependences on initiator concentrations, as observed by linear fits to *k*_obs_ vs. [**1**] (Figure 2; see Figure S47). Thus the overall rate laws are second‐order and the propagation rate constants are *k*_p_=30±3 m ^−1^ min^−1^ (**1**) and 20±1 m ^−1^ min^−1^ (**2**).

**Figure 2:**
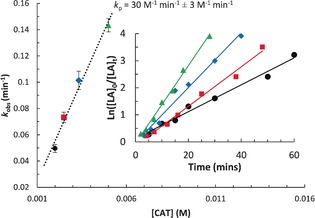
Overlay of semi‐logarithmic first‐order plots for the polymerization of *rac*‐LA initiated by **1**. Reaction conditions: [LA]/[**1**]=500 (black); [LA]/[**1**]=400 (red); [LA]/[1]=300 (blue); [LA]/[**1**]=200 (green), [LA]=1 m, THF, 298 K. Inset: Plot of *k*_obs_ vs. [**1**] for calculation of the propagation rate constant, *k*_p_.

**Table 1:** Polymerization data using **1** and **2** in THF, [LA]=1 m.

Entry	Ini.	*T* [K]	Conv.^[l]^ [%]	*t* [min]	*M*_n_ [kDa]^[m]^	*M*_n_^calc^ [kDa]	*Ð* ^[m]^	*P*_i_^[n]^
1^[a]^	**1**	298	96	60	65.3	69.2	1.15	0.87
2^[b]^	**1**	298	97	48	53.4	55.3	1.17	0.85
3^[c]^	**1**	298	98	40	45.6	42.4	1.20	0.85
4^[d]^	**1**	298	98	28	29.9	28.2	1.18	0.85
5^[e]^	**1**	298	88	30	12.5	12.7	1.22	–
6^[f]^	**1**	298	90	90	22.2	25.9	1.38	–
7^[g]^	**1**	258	96	985	69.8	69.2	1.14	0.92
8^[h]^	**1**	403	56	10	46.2	40.4	2.29	0.66
9^[a]^	**2**	298	80	153	50.8	57.7	1.19	0.75
10^[i]^	**2**	298	93	106	52.0	46.9	1.25	0.72
11^[j]^	**2**	298	97	55	39.4	35.0	1.17	–
Y‐Phos^[k]^		298	80	70 s	105	115	1.08	0.10

[a] 500 equiv. [b] 400 equiv. [c] 300 equiv. [d] 200 equiv. [e] 100 equiv, 0.5 M. [f] Reaction mixture for [e] with an additional 100 equiv of *rac*‐LA added. [g] 500 equiv, 0.75 M. [h] 500 equiv, neat. [i] 350 equiv. [j] 250 equiv. [k] 1000 equiv, 1 m, 1 equiv of *i*PrOH added. [l] Determined by integration of the methine region of the ^1^H NMR spectrum (LA, 4.96–5.04 ppm; PLA, 5.10–5.22 ppm). [m] For initiator **1**: determined by SEC analysis against polystyrene standards in CHCl_3_ using a Mark–Houwink correction factor of 0.58;^11^ for initiator **2**: determined by SEC‐MALLS in THF. [n] Determined by integration of PLA methine tetrads in the ^1^H{^1^H} NMR spectrum using values predicted according to Bernoullian statistics.

Both initiators displayed high degrees of polymerization control as demonstrated by predictable molecular weights (*M_n_*), narrow dispersities, and linear evolutions of *M_n_* values with conversion. By using **2**, slight broadening of distributions (*Ð*=1.20) was observed at higher conversions, and in contrast to using **1**, the distributions remained narrow (*Ð*<1.10) throughout. The analysis of the end groups was conducted using MALDI‐ToF mass spectrometry (catalyst **1**), and showed a single set of peaks assigned to chains with ethoxide end groups. The peaks are separated by 144 amu, which confirms the absence of transesterification side reactions (see Figure S48). Further evidence of control was obtained from a sequential monomer‐addition experiment, in which an additional 100 equivalents of lactide were added to an experiment conducted under conditions as per entries 5 and 6 in Table 1. This set of conditions led to complete conversion and an increase in the molecular weight of the polymer from 12.5 to 22.2 kg mol^−1^ (see Figure S49).

Both catalysts induced high levels of stereocontrol with a strong isotactic bias in resultant polymers, and **1** afforded highly isotactic PLA (*P*_i_=0.85–0.87, 298 K; see Figures S50‐S53). The analysis of defect tetrad resonances in the ^1^H{^1^H} NMR spectrum indicate a chain‐end control mechanism for the isoselectivity,7a, 15 that is, the last inserted lactide unit dictates the selectivity between the two lactide enantiomers. In addition to homonuclear decoupled NMR spectroscopy, differential scanning calorimetry (DSC) confirmed the formation of semi‐crystalline PLA showing a *T*_m_ of 165 °C (see Figure S54), and is consistent with recent literature detailing the variation of *T*_m_ against *P*_i_.5c Given the good rates of polymerizations, it was also feasible to run the polymerizations at low temperature (entry 7, Table 1), and this led to increased isoselectivity with *P*_i_=0.92 at 258 K (see Figure S55). In this case, the DSC analysis showed an increased *T*_m_ of up to 179 °C, thus confirming the formation of stereoblock PLA (Figure 3). A polymerization was also run at 403 K, and at this higher temperature there was a reduction in isoselectivity, although the resultant PLA was moderately isotactic (Table 1, entry 8; see Figure S56). The catalyst **2** showed lower isoselectivity under the standard reaction conditions with *P*_i_=0.75 (see Figures S57 and S58). Thus, it is clear that the bulky *ortho* cumyl substituents in **1** are important to steric protection and direction at the metal center. It is relevant to note that *ortho*‐phenyl substituents were also important in mediating high isoselectivity for aluminium salen complexes, and thus may be considered a useful site for further ligand development studies.7b, 16

**Figure 3:**
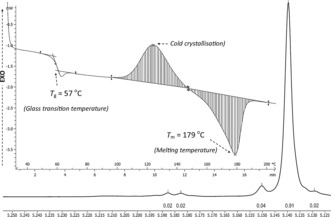
The methine region of the ^1^H{^1^H} NMR spectrum of PLA in CDCl_3_. Reaction conditions: [LA]/[**1**]=500, [LA]=0.75 m, THF, 258 K (*P*_i_=0.92). Inset: Heat flow vs. temperature curve for the aforementioned PLA sample. The curve shows three distinct transitions: *T*_g_, cold crystallization, and *T*_m_.

Another unexpected feature of **2** is that the same ligand coordinated to yttrium leads to a catalyst that is heteroselective (Y‐Phos; Table 1). This selectivity is surprising because in general, a particular ligand scaffold will induce the same tacticity when coordinated to different metals.5n,5o, 12 Analysis of the catalyst structures determined by X‐ray crystallography shows that the yttrium complex has a coordination geometry closer to square‐based pyramidal (*τ*=0.08) and, as expected, shows longer M−N bond lengths [Y–N=2.324(2) Å and 2.375(2) Å vs. In–N=2.203(5) Å and 2.187(5) Å].^12^ One explanation for the different selectivity may be that the yttrium is more tightly coordinated by the ligand, thereby resulting in the less steric hindrance and thus formation of heterotactic PLA. Comparing the performance of **1** against other known isoselective catalysts, particularly of group 13, the best chiral indium salen catalysts show *P*_i_=0.77 (298 K).8a The catalyst **1** is significantly more isoselective and obviates the use of chiral diamines whilst maintaining approximately equivalent rates (Figure 4). Similarly, indium complexes derived upon fluorinated dialkoxy‐diimino salen‐like ligands have afforded moderate *P*_i_ values of up to 0.69.^17^ The best aluminium salen complexes showed *P*_i_=0.97 (368 K).7b However, the rates were slow with *k*_p_=0.054 m ^−1^ min^−1^, which is 500 times slower than **1** (*k*_p_=30.6 m ^−1^ min^−1^).

**Figure 4:**
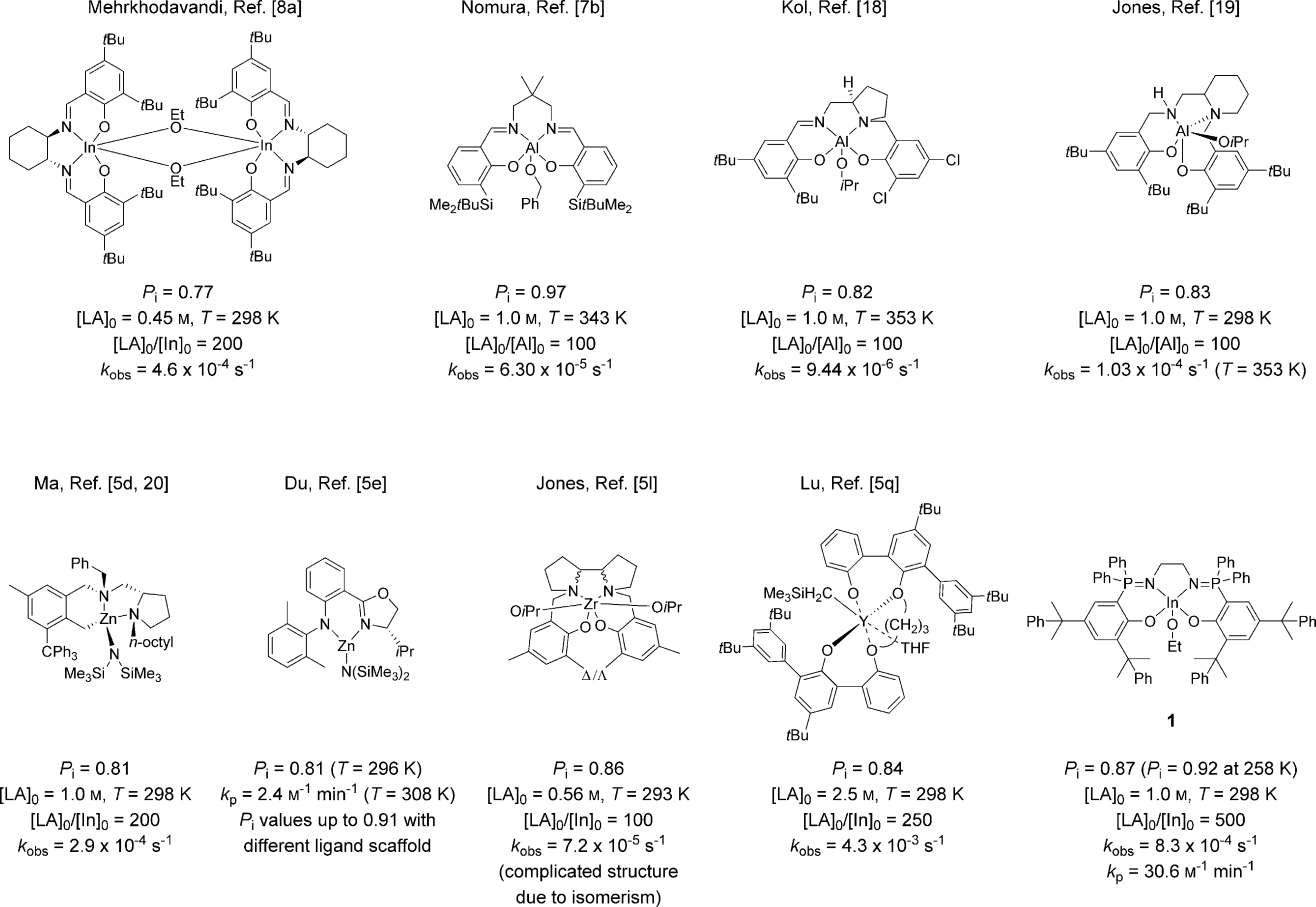
Isoselective initiators for the synthesis of PLA, including this work (bottom right).

In the same study, aluminium/salen complexes, featuring the same *ortho* substituents and an ethylene linker, showed an isoselectivity (*P*_i_=0.85) equivalent to that of **1**, and this direct comparison highlights the potential to deliver both significantly higher rates and isoselectivity relative to those obtained with aluminium/salen catalysts. Aluminium/salalen catalysts incorporating a chiral pyrrolidine ligand showed *P*_i_=0.82 (toluene, 353 K).^18^ Related aluminium/salalen catalysts, incorporating 2‐aminopiperidine, showed *P*_i_=0.83 (CH_2_Cl_2_, 298 K) although the rates were slow.^19^ Thus, **1** shows substantially improved isoselectivity compared to indium/salen catalysts and much higher rates whilst maintaining competitive isoselectivity compared to aluminium/salen catalysts. Considering other metals, by using chiral zinc catalysts, the most selective of which features trityl *ortho* phenyl substituents, gave a maximum value *P*_i_=0.81 (298 K, THF), although the most selective catalyst is approximately four times slower than **1**.5d, 20 Chiral zinc/amido‐oxazolinate catalysts showed *P*_i_=0.91, but are more than 700 times slower with *k*_p_=0.04 m ^−1^ s^−1^ (toluene, 296 K).5e Zirconium/salalen catalysts showed *P*_i_=0.86, but are much slower than **1**.5l Diimino pyrrolide copper catalysts showed lower isoselectivity (*P*_i_=0.70) and lower rates.5g The only comparable catalyst was reported very recently, and is an yttrium bis(phenolate) catalyst showing high rates (*k*_obs_=4.25×10^−3^ s^−1^, toluene, 298 K, [LA]=2.5 m, [catalyst]=10 mm) and isoselectivity (*P*_i_=0.84 at 298 K; *P*_i_=0.90 at 258 K).5q Overall, in comparison with other isoselective catalysts, **1** shows leading performance both in terms of isoselectivity and rate. Furthermore, the catalyst is very well controlled and shows minimal transesterification reactions.

In conclusion, indium phosphasalen catalysts show very good performance and demonstrate the potential for the ligand class and indium in this field of catalysis. The two new catalysts yielded polymers with a strong isotactic bias (*P*_i_=0.87; 298 K) and the incorporation of bulky *ortho* cumyl groups leads to a marked enhancement in isoselectivity. Given the importance of salen‐type ligands in stereoselective catalysis, these phosphasalen ligands and complexes merit exploration as they allow control in stereochemistry without the need for chiral additives/ligands.

## Conflict of interest

The authors declare no conflict of interest.

## Supporting information

As a service to our authors and readers, this journal provides supporting information supplied by the authors. Such materials are peer reviewed and may be re‐organized for online delivery, but are not copy‐edited or typeset. Technical support issues arising from supporting information (other than missing files) should be addressed to the authors.

Supplementary
