# Supplementary material for: Phosphasalen Indium Complexes Showing High Rates and Isoselectivities in rac‐Lactide Polymerizations
Source: Angew Chem Int Ed Engl. 2017 Apr 5;56(19):5277–82. doi: 10.1002/anie.201701745 (PMC5434947; doi:10.1002/anie.201701745)
Supplement: Supplementary file 1 — Supplementary [file ANIE-56-5277-s001.pdf]

Angewandte Chemie
Eine Zeitschrift der Gesellschaft Deutscher Chemiker

Supporting Information

Phosphasalen Indium Complexes Showing High Rates and Isoselectivities in *rac*-Lactide Polymerizations

Dominic Myers, Andrew J. P. White, Craig M. Forsyth, Mark Bown, and Charlotte K. Williams**

anie_201701745_sm_miscellaneous_information.pdf

Supporting Information

Supporting Information

Contents

Experimental 6

Synthetic protocols 8

Reaction schemes 12

Scheme S1. i. NBS (1 eq.), acetonitrile **ii.** n-BuLi (2 eq.), Et_2O **iii.** $ClPPh_2$ (1 eq.) **iv.** Br_2 (1 eq.), CH_2Cl_2 **v.** Bu_3N (1 eq.), ethylene diamine (0.5 eq.). 12

Scheme S2. i. NaH (6 eq.), THF **ii.** $InCl_3$ (1 eq.), **iii.** KOEt (1 eq.). 12

Scheme S3. i. NaH (6 eq.), THF **ii.** $InCl_3$ (1 eq.), **iii.** KO^tBu (1 eq.). 12

Figure S1. ^1H NMR spectrum of compound $\mathbf{L^1}$ in $CDCl_3$. 13

Figure S2. ^{31}P NMR spectrum of compound $\mathbf{L^1}$ in $CDCl_3$. 13

Figure S3. ^{13}C NMR spectrum of compound $\mathbf{L^1}$ in $CDCl_3$. (Bottom left inset) DEPT 135 spectrum. (Top right inset) DEPT 90 spectrum. 14

Figure S4. ^1H NMR spectrum of compound **1** in C_6D_6. 14

Figure S5. ^{31}P NMR spectrum of compound **1** in C_6D_6 15

Figure S6. ^{13}C NMR spectrum of compound **1** in C_6D_6. (Bottom left inset) DEPT 90 spectrum. (Top right inset) DEPT 135 spectrum. 15

Figure S7. (Left) Electrospray (ES) ionisation mass spectrum (MS) of compound **1**. (Right) Close-up zoom of the molecular ion peak in the ES-MS of compound **1**. The molecular ion peak is identified as $[M\text{-}OEt]^{+\cdot}$ 16

Figure S8. ^1H NMR spectrum of compound **2** in C_6D_6 17

Figure S9. ^{31}P NMR spectrum of compound **2** in C_6D_6 17

Figure S10. ^{13}C NMR spectrum of compound **2** in C_6D_6. (Bottom left inset) DEPT 135 spectrum. (Top right inset) DEPT 90 spectrum. 18

Figure S11. ^1H ROESY NMR spectrum of compound **1** in THF-d_8 18

Figure S12. ^1H VT NMR spectrum of compound **1** in toluene-d_8 19

Figure S13. ^1H VT NMR spectrum of compound **1** (zoom on aromatic resonances) in toluene-d_8 19

Figure S14. ^1H VT NMR spectrum of compound **1** (zoom on ethylene backbone resonances) in toluene-d_8 20

Figure S15. ^1H VT NMR spectrum of compound **1** (zoom on ortho-cumyl methyl group resonances) in toluene-d_8 20

Figure S16. The crystal structure of the C_i-symmetric complex **1** (50% probability ellipsoids). 21

Figure S17. The crystal structure of **2**. 22

Figure S18. The crystal structure of **2** (50% probability ellipsoids). 22

Table S1. Selected bond lengths (Å) and angles (°) for compound **1**. 23

Table S2. Selected bond lengths (Å) and angles (°) for compound **2**. 23

Figure S19. ^{1}H DOSY NMR of compound **1** in benzene-d_6 at 298 K.. 24

Figure S20. ^{1}H DOSY NMR of compound **1** in toluene-d_8 at 298 K.. 24

Figure S21. ^{1}H DOSY NMR of compound **1** in THF-d_8 at 298 K... 25

Figure S22. ^{1}H DOSY NMR of compound **2** in THF-d_8 at 298 K... 25

Figure S23. Conversion vs time plot for the polymerisation of rac-LA mediated by compound **1**. Conditions: [LA]/[**1**] = 500, [LA] = 1 M, THF, 298 K.. 26

Figure S24. Plot of the molecular weight and polydispersity vs conversion for the polymerisation of rac-LA mediated by compound **1**. Conditions: [LA]/[**1**] = 500, [LA] = 1 M, THF, 298 K. 26

Figure S25. Overlay of SEC traces for the polymerisation of rac-LA mediated by **1**. Conditions: [LA]/[**1**] = 500, [LA] = 1 M, THF, 298 K 26

Figure S26. Conversion vs time plot for the polymerisation of rac-LA mediated by compound **1**. Conditions: [LA]/[**1**] = 400, [LA] = 1 M, THF, 298 K.. 27

Figure S27. Plot of the molecular weight and polydispersity vs conversion for the polymerisation of rac-LA mediated by compound **1**. Conditions: [LA]/[**1**] = 400, [LA] = 1 M, THF, 298 K. 27

Figure S28. Overlay of SEC traces for the polymerisation of rac-LA mediated by **1**. Conditions: [LA]/[**1**] = 400, [LA] = 1 M, THF, 298 K. 27

Figure S29. Conversion vs time plot for the polymerisation of rac-LA mediated by compound **1**. Conditions: [LA]/[**1**] = 300, [LA] = 1 M, THF, 298 K.. 28

Figure S30. Plot of the molecular weight and polydispersity vs conversion for the polymerisation of rac-LA mediated by compound **1**. Conditions: [LA]/[**1**] = 300, [LA] = 1 M, THF, 298 K. 28

Figure S31. Overlay of SEC traces for the polymerisation of rac-LA mediated by **1**. Conditions: [LA]/[**1**] = 300, [LA] = 1 M, THF, 298 K. 28

Figure S32. Conversion vs time plot for the polymerisation of rac-LA mediated by compound **1**. Conditions: [LA]/[**1**] = 200, [LA] = 1 M, THF, 298 K.. 29

Figure S33. Plot of the molecular weight and polydispersity vs conversion for the polymerisation of rac-LA mediated by compound **1**. Conditions: [LA]/[**1**] = 200, [LA] = 1 M, THF, 298 K. 29

Figure S34. Overlay of SEC traces for the polymerisation of rac-LA mediated by **1**. Conditions: [LA]/[**1**] = 200, [LA] = 1 M, THF, 298 K. 29

Figure S35. Conversion vs time plot for the polymerisation of rac-LA mediated by compound **2**. Conditions: [LA]/[**2**] = 500, [LA] = 1 M, THF, 298 K.. 30

Figure S36. Plot of the molecular weight and polydispersity vs conversion for the polymerisation of rac-LA mediated by compound **2**. Conditions: [LA]/[**2**] = 500, [LA] = 1 M, THF, 298 K. 30

Figure S37. Overlay of SEC traces for the polymerisation of rac-LA mediated by **2**. Conditions: [LA]/[**2**] = 500, [LA] = 1 M, THF, 298 K. 30

Figure S38. Conversion vs time plot for the polymerisation of rac-LA mediated by compound **2**. Conditions: [LA]/[**2**] = 350, [LA] = 1 M, THF, 298 K.. 31

Figure S39. Plot of the molecular weight and polydispersity vs conversion for the polymerisation of rac-LA mediated by compound **2**. Conditions: [LA]/[**2**] = 350, [LA] = 1 M, THF, 298 K 31

Figure S40. Overlay of SEC traces for the polymerisation of rac-LA mediated by **2**. Conditions: [LA]/[**2**] = 350, [LA] = 1 M, THF, 298 K. 31

Figure S41. Conversion vs time plot for the polymerisation of rac-LA mediated by compound **2**. Conditions: [LA]/[**2**] = 250, [LA] = 1 M, THF, 298 K. 32

Figure S42. Plot of the molecular weight and polydispersity vs for the polymerisation of rac-LA mediated by compound **2**. Conditions: [LA]/[**2**] = 250, [LA] = 1 M, THF, 298 K. 32

Figure S43. Overlay of SEC traces for the polymerisation of rac-LA mediated by **2**. Conditions: [LA]/[**2**] = 250, [LA] = 1 M, THF, 298 K. 32

Figure S44. Semi-logarithmic first-order plot for the polymerisation of rac-LA mediated by compound **2**. Conditions: [LA]/[**2**] = 500, [LA] = 1 M, THF, 298 K. 33

Figure S45. Semi-logarithmic first-order plot for the polymerisation of rac-LA mediated by compound **2**. Conditions: [LA]/[**2**] = 350, [LA] = 1 M, THF, 298 K. 33

Figure S46. Semi-logarithmic first-order plot for the polymerisation of rac-LA mediated by compound **2**. Conditions: [LA]/[**2**] = 250, [LA] = 1 M, THF, 298 K. 33

Figure S47. Plot of k_{obs} vs [**2**] for the polymerisation of rac-LA mediated by compound **2**. Conditions: [LA] = 1 M, THF, 298 K. 34

Figure S48. MALDI-ToF spectrum obtained from the polymerisation of rac-LA mediated by **1**. Conditions: [LA]/[**1**] = 500, [LA] = 0.75 M, THF, 244 K. Conv. = 8 %, time = 4 h, M_n = 6.5 kg mol^{-1} (SEC), PDI = 1.08 (SEC), M_n = 5.4 kg mol^{-1} (MALDI), PDI = 1.03 (MALDI), M_n^{theo} = 5.8 kg mol^{-1} 34

Figure S49. Overlay of SEC traces for the polymerisation of 100 eq. + 100 eq. of rac-LA with **1**. Conditions: [LA] = 0.5 M, THF, 298 K 35

Figure S50. Deconvoluted homonuclear-decoupled ^{1}H NMR spectrum of the methine region of PLA obtained with **1** in $CDCl_3$; P_i values determined by evaluation of all tetrads. (Table 1, entry 1 in publication). 35

Figure S51. Deconvoluted homonuclear-decoupled ^{1}H NMR spectrum of the methine region of PLA obtained with **1** in $CDCl_3$; P_i values determined by evaluation of all tetrads. (Table 1, entry 2 in publication). 36

Figure S52. Deconvoluted homonuclear-decoupled ^{1}H NMR spectrum of the methine region of PLA obtained with **1** in $CDCl_3$; P_i values determined by evaluation of all tetrads. (Table 1, entry 3 in publication). 36

Figure S53. Deconvoluted homonuclear-decoupled ^{1}H NMR spectrum of the methine region of PLA obtained with **1** in $CDCl_3$; P_i values determined by evaluation of all tetrads. (Table 1, entry 4 in publication). 37

Figure S54. Heat flow vs temperature curve for a PLA sample obtained from **1**. (Table 1, entry 1 in publication). 37

Figure S55. Deconvoluted homonuclear-decoupled ^{1}H NMR spectrum of the methine region of PLA obtained with **1** in $CDCl_3$; P_i values determined by evaluation of all tetrads. (Table 1, entry 7 in publication). 38

Figure S56. Deconvoluted homonuclear-decoupled ^{1}H NMR spectrum of the methine region of PLA obtained with **1** in $CDCl_3$; P_i values determined by evaluation of all tetrads. (Table 1, entry 8 in publication). 38

Figure S57. Deconvoluted homonuclear-decoupled ^{1}H NMR spectrum of the methine region of PLA obtained with **2** in $CDCl_3$; P_i values determined by evaluation of all tetrads. (Table 1, entry 9 in publication). 39
Figure S58. Deconvoluted homonuclear-decoupled ^{1}H NMR spectrum of the methine region of PLA obtained with **2** in $CDCl_3$; P_i values determined by evaluation of all tetrads. (Table 1, entry 10 in publication). 39
Bibliography........ 40

Experimental Section

All solvents and reagents were obtained from commercial sources (Aldrich, VWR and Strem) and used as received unless stated otherwise. For organometallic reactions, tetrahydrofuran (THF), hexane and cyclohexane solvents were refluxed over sodium/benzophenone and stored over activated molecular sieves under nitrogen. Diethylether and toluene used for ligand preparation or as a recrystallisation solvent were taken directly from an MBraun MB-SPS 800 Solvent Purification system and stored over activated molecular sieves. THF-d_8, toluene-d_8 and benzene-d_6 deuterated solvents were distilled over sodium/benzophenone and stored over activated molecular sieves under nitrogen. All dry solvent and reagents were degassed by several freeze-pump-thaw cycles before being stored under nitrogen. Unless stated otherwise, all ligand preparation reactions performed under an inert atmosphere were performed using a double-manifold Schlenck vacuum line under nitrogen. All three-step metallation reactions were performed in a nitrogen-filled glovebox ($N_2 < 0.1$ ppm, $O_2 < 0.1$ ppm). The phosphasalen ligand, $\mathbf{L^2}$, was synthesised according to literature protocols.[1] *Rac*-LA was recrystallized twice from anhydrous toluene under a nitrogen atmosphere before twice being sublimed and stored under nitrogen before use.

NMR Spectroscopy: ^1H, ^{13}C, ^{31}P and 2D NMR (COSY, HSQC, HMBC) spectra were recorded using a Bruker AV 400 MHz spectrometer at 298 K. $^1H\{^1H\}$ NMR and variable temperature NMR studies were also recorded using a Bruker AV 400 MHz spectrometer. ROESY and DOSY NMR experiments were recorded using a Bruker AV 500 MHz spectrometer. The tacticity of polymers was determined from its homonuclear-decoupled ^1H NMR spectrum, using deconvolution techniques from MestReNova v. 8.0.0. The overall isoselectivity, P_i, was determined by taking an average of tetrad integrals predicted from Bernoullian statistics (equations given in section on 'data fitting'). The following abbreviations are used in the report of spectra: br, broad; s, singlet; d, doublet; dd, doublet of doublets; t, triplet; q, quartet; m, multiplet.

ESI-MS: ESI-MS spectra were collected by Carl Braybrook and mass spectrometric analyses were performed on a Thermo Scientific Q Exactive mass spectrometer fitted with a HESI-II ion source. Positive *and/or negative* ion electrospray mass spectra were recorded in an appropriate mass range set for *140,000* mass resolution. The probe was used with 0.3 ml/min flow of *solvent*. The nitrogen nebulizing/desolvation gas used for vaporization was heated to *350*°C in these experiments. The sheath gas flow rate was set to *35* and the auxiliary gas flow rate to *25* (both arbitrary units). The spray voltage was *3.0*kV and the capillary temperature was *300*°C.

MALDI-TOF MS: MALDI ToF analysis was carried out by Carl Braybrook with spectra run on a Bruker Autoflex III MALDI TOF/TOF mass spectrometer using positive ion in reflectron mode. Laser power was optimized and set to 40. The matrix employed was DCTB at a concentration of 10 mg ml^{-1} in $CHCl_3$/EtOH. 10 µL of this solution was co-applied with the 1 µL KTFA and 1 ul of the analyte (1 mg mL^{-1}; $CHCl_3$/EtOH) to a steel target plate for analysis. M_n, M_w and Đ values were calculated using the Bruker PolyTools Synthetic Polymer Analysis software package version 1.18.

X-ray diffraction: X-ray diffraction data was collected using a Bruker APEX II CCD diffractometer for **1** and an Agilent Xcalibur 3 E diffractometer for **2**. Further crystallographic data can be found in Figs. S16-S18 and Tables S1-S2.

Elemental analysis: Elemental analysis (C,H,N) for all novel ligands and complexes were were carried out by Mr Stephen Boyer at London Metropolitan University except ligand $\mathbf{L^1}$, which was analysed by Mr Bob McAllister from the Campbell Microanalytical Laboratory at the University of Otago, with a standard error of ±0.3%.

Gel permeation chromatography: For polymers obtained by ROP of *rac*-LA initiated by compound **1**, Four Agilent PL-Gel columns (3 x PL-Gel Mixed C (5 µm) and 1 x PL-Gel Mixed E (3 µm) columns) were used in series, with HPLC grade chloroform (amylene-stabilised) as the eluent, at a flow rate of 1 mL min^{-1}, on a Waters Alliance system equipped with an Alliance 2695 Separation Module at 30 °C. Polymer number-average molecular weight (M_n) and polydispersity index (M_w/M_n; PDI) were calibrated against low dispersity polystyrene standards using a 3rd order polynomial fit, linear across molar mass ranges. As molar masses were reported as polystyrene equivalents, a Mark-Houwink correction factor of 0.58 was used.[2] For polymers obtained by ROP of *rac*-LA initiated by compound **2**, PLA molecular weight information (M_n and PDI) were determined by gel permeation chromatography, equipped with multi-angle laser light scattering (GPC-MALLS). Two Mixed Bed PSS SDV linear S columns were used in series, with THF as the eluent, at a flow rate of 1 mL min^{-1}, on a Shimadzu LC-20AD instrument at 40 °C. The light scattering detector was a triple-angle detector (Dawn 8, Wyatt Technology), and the data was analysed using Astra Version 6.1. The refractive angle increment for polylactide (dn/dc) in THF was 0.040 mL g^{-1}.[3] All polymers were filtered prior to analysis.

DSC: Differential scanning calorimetry was performed on a DSC 821^e using 100 µl aluminium crucibles (Mettler Toledo) under nitrogen. For each sample, approximately 5 - 6 mg of material was heated at a rate of 5 K min^{-1}. The glass transition temperature, T_g, and the melting temperature, T_m, were determined using the software pack Star^e (Mettler Toledo, version 14.0). The T_g was determined by the peak onset and the T_m, the peak maximum was used.

Synthetic protocols

Compound S1

At 273 K, *N*-bromosuccinimide (5.4 g, 30.3 mmol) was added slowly to a solution of 2,4-di-cumylphenol (10.0 g, 30.3 mmol) in acetonitrile (150 mL) under continuous stirring. The reaction mixture was allowed to warm to 298 K and left stirring overnight for 16 h. To the afforded orange solution, a saturated aqueous solution of sodium sulphite (~10 mL) was then added, inducing the formation of a white precipitate. The mixture was then filtered, the white solid separated and the filtrate extracted with petroleum ether (2 x 50 mL). The organic layer was then dried ($MgSO_4$) and the solvent removed *in vacuo*. The product was isolated as a yellow oil (7.5 g, 61 %).

^{1}H NMR (400 MHz, $CDCl_3$, 298 K) δ (ppm): 7.34-7.17 (m, 12H, *Ph*), 5.17 (s, 1H, O*H*), 1.70 (s, 6H, $C^{IV}(CH_3)_2Ph$), 1.62 (s, 6H, $C^{IV}(CH_3)_2Ph$).

Compound S2

Under a dry atmosphere of N_2, a solution of 2-bromo-3,5-di-*tert*-cumylphenol (4.0 g, 9.8 mmol) in Et_2O (~100 mL) was cooled to 195 K before subsequently, a solution of *n*-butyl lithium (1.5 M in hexanes, 13.0 mL, 19.6 mmol) was added affording a white suspension. The reaction mixture under continuous stirring was allowed to warm to 298 K and left for 3 h. The resultant turbid white suspension was cooled back down to 195 K and chlorodiphenylphosphine (1.75 mL, 9.8 mmol) was added to the solution. The mixture was then left to warm to 298 K and left stirring for 18 h. The afforded thick white suspension was then washed with aqueous solutions of $Na_2H_2PO_4$ (0.1 M, 2 x 100 mL) and the organic layer separated. The solution was then dried ($MgSO_4$), methanol (20 mL) added and reduced *in vacuo* until approximately 20 mL of solvent remained. The solution was then left until a white solid precipitated from the yellow solution. The solid was isolated by filtration, washed with MeOH (2 x 20 mL) and dried under vacuum. The product was isolated as a white crystalline solid (3.3 g, 6.41 mmol, 66 %).

^{1}H NMR (400 MHz, $CDCl_3$, 298 K) δ (ppm): 7.31 (d, $^{4}J_{H,H}$ = 2.3 Hz, 1H, C_bH), 7.30-7.12 (m, 20H, $C^{IV}(CH_3)_2Ph$ + PPh_2), 6.54 (dd, $^{3}J_{P,H}$ = 5.3 Hz, $^{4}J_{H,H}$ = 2.3 Hz, 1H, C_dH), 5.11 (d, $^{4}J_{P,H}$ = 1.9 Hz, 1H, O*H*), 1.61 (s, 6H, $C^{IV}(CH_3)_2$), 1.53 (s, 6H, $C^{IV}(CH_3)_2$); $^{13}C\{^{1}H\}$ NMR (100 MHz, $CDCl_3$, 298 K) δ (ppm): 154.1 (d, $^{2}J_{P,C}$ = 18.8 Hz, C^{IV}-OH) 150.8 (s, $C_{a,c}^{IV}$-$C^{IV}(CH_3)_2$-C^{IV}), 149.0 (s, $C_{a,c}^{IV}$-$C^{IV}(CH_3)_2$-C^{IV}), 142.6 (s, $C_{a,c}^{IV}$), 136.3 (d, $J_{P,C}$ = 7.1 Hz, *Ph*), 134.7 (s, $C_{a,c}^{IV}$), 133.7 (d, $J_{P,C}$ = 18.9 Hz, *Ph*), 131.1 (s, C_dH), 128.9 (s, *Ph*), 128.7 (s, *Ph*), 128.5 (d, $J_{P,C}$ = 7.5 Hz, *Ph*), 128.0 (s, *Ph*), 126.8 (s, *Ph*), 126.7 (s, C_bH), 126.0 (d, $^{1}J_{P,C}$ = 101.7 Hz, C^{IV}-P), 42.6 (s, $C^{IV}(CH_3)_2$), 42.2 (s, $C^{IV}(CH_3)_2$), 30.9 (s, $C^{IV}(CH_3)_2$), 29.7 (s, $C^{IV}(CH_3)_2$); $^{31}P\{^{1}H\}$ NMR (160 MHz, $CDCl_3$, 298 K) δ (ppm): -20.5 (s, *P*). Anal. Calc. ($C_{36}H_{35}OP$): C, 84.02; H, 6.86. Found: C, 83.86; H, 6.76.

Compound L^1

At 195 K, bromine (149 µL, 2.91 mmol) was added to a solution of **S2** (1.50 g, 2.91 mmol) in dichloromethane (50 mL). The solution was then allowed to warm to 298 K and left to stir for 2 h. The solution was then cooled again to 195 K, after which, tributylamine (694 µL, 2.91 mmol) was added, followed by ethylenediamine (97 µL, 1.46 mmol). The solution was then allowed to warm to 298 K and left stirring for 16 h overnight affording a pale yellow turbid suspension The solvent was then removed *in vacuo* leaving behind a yellow gelatinous product. To the crude mixture, tetrahydrofuran (15 mL) was added followed by petroleum ether (2 mL) under vigorous stirring. After 4 h, the afforded white

suspension was filtered and washed with further portions of tetrahydrofuran (3 x 5 mL). The product was then dried and isolated as a white solid (1.4 g, 77 %).

^{1}H NMR (400 MHz, $CDCl_3$, 298 K) δ (ppm): 7.59 (d, $^4J_{H,H}$ = 2.1 Hz, 2H, C_bH), 7.58-7.54 (m, 4H, P*Ph*$_2$), 7.53-7.46 (m, 8H, P*Ph*$_2$), 7.44-7.37 (m, 8H, P*Ph*$_2$), 7.29-7.07 (m, 20H, $C^{IV}(CH_3)_2Ph$), 6.98 (m, 2H, N*H*), 6.75 (dd, $^3J_{P,H}$ = 15.5 Hz, $^4J_{H,H}$ = 2.1 Hz, 2H, C_dH), 6.19 (m, 2H, O*H*), 3.22 (m, 4H, $(CH_2)_2$), 1.64 (s, 12H, $C_a^{IV}(CH_3)_2Ph$), 1.57 (s, 12H, $C_c^{IV}(CH_3)_2Ph$); $^{13}C\{^1H\}$ NMR (100 MHz, $CDCl_3$, 298 K) δ (ppm): 154.8 (s, C^{IV}-OH), 149.3 (s, C_c^{IV}), 147.8 (s, C_a^{IV}), 134.2 (d, $J_{P,C}$ = 2.2 Hz, P*Ph*$_2$), 133.4 (d, $J_{P,C}$ = 11.9 Hz, P*Ph*$_2$), 133.1 (s, C_bH), 131.1 (d, C_dH, $^2J_{P,C}$ = 10.7 Hz), 129.6 (d, $J_{P,C}$ = 12.0 Hz, P*Ph*$_2$), 129.1 (s, $C^{IV}(CH_3)_2Ph$), 128.3 (s, $C^{IV}(CH_3)_2Ph$), 126.9 (s, $C^{IV}(CH_3)_2Ph$), 126.8 (s, $C^{IV}(CH_3)_2Ph$), 126.1(s, $C^{IV}(CH_3)_2Ph$), 126.0(s, $C^{IV}(CH_3)_2Ph$), 121.7 (d, $^1J_{P,C}$ = 104.4 Hz, P-C^{IV}), 43.7 (d, $^2J_{P,C}$ = 7.8 Hz, $(CH_2)_2$) 43.1 (s, C_c^{IV}-$C^{IV}(CH_3)_2$), 42.3 (s, C_a^{IV}-$C^{IV}(CH_3)_2$), 30.8 (s, C_c^{IV}-$C^{IV}(CH_3)_2$), 30.0 (s, C_a^{IV}-$C^{IV}(CH_3)_2$); $^{31}P\{^1H\}$ NMR (160 MHz, $CDCl_3$, 298 K) δ (ppm): 39.6 (s, *P*). Anal. Calc. ($C_{74}H_{76}Br_2N_2O_4P_2$): C, 71.27; H, 6.14; N, 2.25. Found: C, 70.88; H, 6.21; N, 2.26.

Compound 1

Sodium hydride (30.8 mg, 1.28 mmol) was added to a slurry of ligand $\mathbf{L^1}$ (200 mg, 0.16 mmol) in THF (10 mL) under continuous stirring at 298 K. After 16 h, the afforded turbid white suspension was checked by $^{31}P\{^1H\}$ NMR spectroscopy, which showed clean formation of the deprotonated species with a singlet at 19.1 ppm. The solution was then filtered to remove insoluble sodium salts before subsequent addition of indium trichloride (35.5 mg, 0.16 mmol) and the reaction stirred for another 16 h at 298 K. The $^{31}P\{^1H\}$ NMR spectrum of the afforded cloudy solution was then checked for clean formation of an indium chloride complex confirmed by a singlet at 40.8 ppm. Potassium ethoxide (13.5 mg, 0.16 mmol) was then added to the reaction mixture and stirring continued for a further 12 h at 298 K. The $^{31}P\{^1H\}$ NMR spectrum was again checked and indicated clean formation of the indium alkoxide species with a singlet at 39.5 ppm. The cloudy solution was then filtered and the solvent removed *in vacuo*. Finally, the residue was dissolved in cyclohexane (5 mL) and left to crystallise, affording the product as colourless crystals (63 mg, 31 %).

^{1}H NMR (400 MHz, C_6D_6, 298 K) δ (ppm): 7.61-7.21 (m, 14H, C_bH + P*Ph*$_2$ + $C_{a,c}^{IV}(CH_3)_2Ph$), 7.20-6.83 (m, 28H, P*Ph*$_2$ + $C_{a,c}^{IV}(CH_3)_2Ph$), 6.39 (d, $^3J_{P,H}$ = 17.3 Hz, 2H, C_dH), 4.29 (q, $^3J_{H,H}$ = 6.7 Hz, 2H, O-CH_2CH_3), 3.06-2.05 (m, br, 4H, $(CH_2)_2$), 2.47-1.93 (m, br, 12H, $C_a^{IV}(CH_3)_2Ph$), 1.41 (s, 12H, $C_c^{IV}(CH_3)_2Ph$), 1.35 (t, $^3J_{H,H}$ = 6.7 Hz, 2H, CH_2CH_3); $^{13}C\{^1H\}$ NMR (100 MHz, C_6D_6, 298 K) δ (ppm): 181.1 (s, C_a^{IV}-C^{IV}-O-In), 170.6 (s, C_a^{IV}), 152.2 (s, P*Ph*$_2$), 151.2 (s, C_dH-C^{IV}-P), 142.7 (d, $J_{P,C}$ = 9.3 Hz, *Ph*), 135.7 (s, C_c^{IV}), 134.0 (m, *Ph*), 132.8 (s, C_bH + *Ph*), 129.1 (s, C_dH), 128.7 (d, $J_{P,C}$ = 12.7 Hz, *Ph*), 128.4 (s, *Ph*), 128.2 (s, *Ph*), 127.9 (s, *Ph*), 127.6 (s, *Ph*), 127.0 (s, *Ph*), 125.5 (s, *Ph*), 125.0 (s, *Ph*), 62.2 (s, CH_2CH_3), 46.8 (m, $(CH_2)_2$), 44.4 (C_a^{IV}-$C^{IV}(CH_3)_2Ph$), 42.4 (C_c^{IV}-$C^{IV}(CH_3)_2Ph$), 30.8 (C_a^{IV}-$C^{IV}(CH_3)_2Ph$ + (C_c^{IV}-$C^{IV}(CH_3)_2Ph$), 28.5 (C_a^{IV}-$C^{IV}(CH_3)_2Ph$), 22.8 (s, CH_2CH_3); $^{31}P\{^1H\}$ NMR (160 MHz, C_6D_6, 298 K) δ (ppm): 39.6 (s, *P*). HRMS (ESI-TOF) m/z: [M-OEt]$^{+\cdot}$ Calcd for $C_{74}H_{72}InN_2O_2P_2$ 1197.4108; Found 1197.4108.

Compound 2

Sodium hydride (28.8 mg, 1.20 mmol) was added to a slurry of ligand $\mathbf{L^2}$ (200 mg, 0.20 mmol) in THF (10 mL) under continuous stirring at 298 K. After 16 h, the afforded turbid white suspension was checked by $^{31}P\{^1H\}$ NMR spectrum, which showed clean formation of the deprotonated species by a singlet at 25.4 ppm. The insoluble sodium salts were then removed from the solution by centrifugation before subsequent addition of indium trichloride (44.3 mg, 0.20 mmol) and the reaction stirred for another 16 h at 298 K. The $^{31}P\{^1H\}$ NMR spectrum of the afforded cloudy solution was then checked

for clean formation of an indium chloride complex confirmed by a singlet at 41.9 ppm. Potassium *tert*-butoxide (22.5 mg, 0.20 mmol) was then added to the reaction mixture and stirring continued for a further 12 h at 298 K. The $^{31}P\{^1H\}$ NMR spectrum was again checked and indicated clean formation of the indium alkoxide species with a singlet at 40.3 ppm. The cloudy solution was then centrifuged to remove all remaining insoluble salts and the solvent removed *in vacuo*. Finally, the residue was washed with hexane (5 mL) inducing precipitation of a white solid which was then isolated and dried (90 mg, 44 %).

^1H NMR (400 MHz, C_6D_6, 298 K) δ (ppm): 7.79-7.72 (m, 6H, C_bH + PPh_2), 7.74 (d, $^4J_{H,H}$ = 2.4 Hz, C_bH, 2H), 7.50-7.42 (m, 4H, PPh_2), 7.11-7.05 (m, 6H, PPh_2), 7.00-6.85 (m, 6H, PPh_2), 6.59 (dd, $^3J_{P,H}$ = 17.0 Hz, $^4J_{H,H}$ = 2.3 Hz, 2H, C_dH), 3.11 (m, br, 2H, $(CH_2)_2$), 2.81 (m, br, 2H, $(CH_2)_2$), 1.97 (s, 18H, C_a^{IV}-$C^{IV}(CH_3)_3$), 1.45 (s, 9H, In-O-$C^{IV}(CH_3)_3$), 1.12 (s, 18H, C_c^{IV}-$C^{IV}(CH_3)_3$); $^{13}C\{^1H\}$ NMR (100 MHz, C_6D_6, 298 K) δ (ppm): 170.7 (s, C_{IV}-O-In), 142.8 (d, $^3J_{P,C}$ = 10.6 Hz, C_a^{IV}), 139.4 (d, $^3J_{P,C}$ = 13.7 Hz, C_c), 134.0 (d, $J_{P,C}$ = 9.3 Hz, *Ph*), 132.7 (d, $J_{P,C}$ = 9.3 Hz, *Ph*), 131.9 (s, *Ph*), 131.1 (s, *Ph*), 128.9 (s, *Ph*), 128.5 (s, *Ph*), 128.4 (d, $^4J_{P,C}$ = 6.1 Hz, C_bH), 127.5 (s, *Ph*), 126.4 (d, $^2J_{P,C}$ = 13.4 Hz, C_dH), 69.0 (s, O-$C^{IV}(CH_3)_3$), 46.5 (s, $(CH_2)_2$), 36.6 (s, C_a-$C^{IV}(CH_3)_3$), 35.2 (s, O-$C^{IV}(CH_3)_3$), 34.1 (s, C_c^{IV}-$C^{IV}(CH_3)_3$), 31.6 (s, C_c^{IV}-$C^{IV}(CH_3)_3$), 30.9 (s, C_a^{IV}-$C^{IV}(CH_3)_3$); $^{31}P\{^1H\}$ NMR (160 MHz, C_6D_6, 298 K) δ (ppm): 40.3 (s, *P*). Anal. Calc. ($C_{58}H_{73}InN_2O_3P_2$): C, 68.10; H, 7.19; N, 2.74. Found: C, 67.87; H, 7.32; N, 2.82.

Typical polymerisation procedure (ambient): In a nitrogen-filled glovebox, a silanised vial was charged with *rac*-LA (232 mg, 1.6 mmol) and dissolved in THF (1.3 mL). A stock solution of the initiator (0.3 mL, 0.011 M) was injected into the solution of monomer such that the overall concentration of lactide was 1 M and the initiator, 2 mM. Aliquots were taken inside the glovebox at specific time intervals and precipitated in hexane (~ 1 mL). The aliquots were then removed from the glovebox and left to evaporate. The crude product was analysed by ^1H NMR (and $^1H\{^1H\}$ NMR where applicable) spectroscopy and GPC. Polymer conversion yields were determined by relative integration of the methine proton resonances for the polymer and monomer. The tacticity (P_i or P_s) was determined by integration of the tetrad resonances in the $^1H\{^1H\}$ decoupled NMR of the polymer. In the cases of high P_i values ($P_i \geq 0.87$), the polymers were purified and their thermal properties analysed by DSC.

Typical polymerisation procedure (low temperature): In a nitrogen-filled glovebox, a silanised vial was charged with *rac*-LA (288 mg, 2.0 mmol) and dissolved in THF (1.66 mL). A stock solution of the initiator (1.0 mL, 0.004 M) was injected into the solution of monomer such that the overall concentration of lactide was 0.75 M and the initiator 1.5 mM. The vial was then sealed, removed from the glovebox placed into a (-15 °C) freezer equipped with a stirring functionality. After the allotted reaction time, the vial was removed from the freezer and quenched into hexane (5 mL) and the solvent allowed to evaporate. The sample was then analysed used the methods described above.

Typical polymerisation procedure (high temperature– melt): In a nitrogen-filled glovebox, a silanised vial was loaded with *rac*-LA (191 mg, 1.3 mmol) and the initiator (3.5 mg, 0.0026 mmol). The vial was then sealed, removed from the glovebox and placed into an oil bath set at 130 °C. The reaction was stirred at 130 °C for 10 mins before the vial was exposed to air and a few drops of $CDCl_3$ added to destroy the active catalyst. The mixture was then analysed using the methods described above.

Silanisation procedure: Hot vials heated in an oven at 105 °C were rinsed several times in a solution of dichlorodimethylsilane in dichloromethane (0.8 M, 10 mL) before being left to dry in an oven prior to use.

Polymer purification (for DSC): The crude polymer mixture was dissolved in THF (~2 mL) and injected into a solution of methanol (~5-10 mL) to precipitate the polymer. The mixture was then centrifuged and the filtrate discarded. This was repeated twice more and the ^{1}H NMR subsequently checked to verify the absence of monomer signals. Finally, the polymer was dissolved in dichloromethane (~3 mL) and rapidly filtered through a small pad of silica. The solvent was removed *in vacuo* and dried both under vacuum and by oven, set to 40 °C.

Stereocontrol determination in polymers: The tacticity of a polymer was determined from its homonuclear-decoupled ^{1}H NMR spectrum, which displayed 5 peaks, each corresponding to a tetrad resonance and reflects the relative stereochemistry along the polymer chain, see Figs. S50-S53 and S55-S58 for ^{1}H{^{1}H} spectra. Peak deconvolution (with residuals minimised), was carried out using MestReNova v. 8.0.0, and was used to determine tetrad integrals. The P_i values were determined from each of the tetrad integrals using the probabilities predicted from Bernoullian statistics.[4] The equations used are given below:

Tetrad	Probability
**sis**	$\frac{P_s^2}{2}$
**sii**	$\frac{P_i P_s}{2}$
**iis**	$\frac{P_i P_s}{2}$
**iii**	$P_i^2 + \frac{P_i P_s}{2}$
**isi**	$\frac{P_s^2 + P_i P_s}{2}$

P_i values were then determined for each tetrad and were highly reproducible, thus the P_i reported is an average of multiple runs and are reported with an approximate standard error in the mean of ±3 %.

Reaction schemes

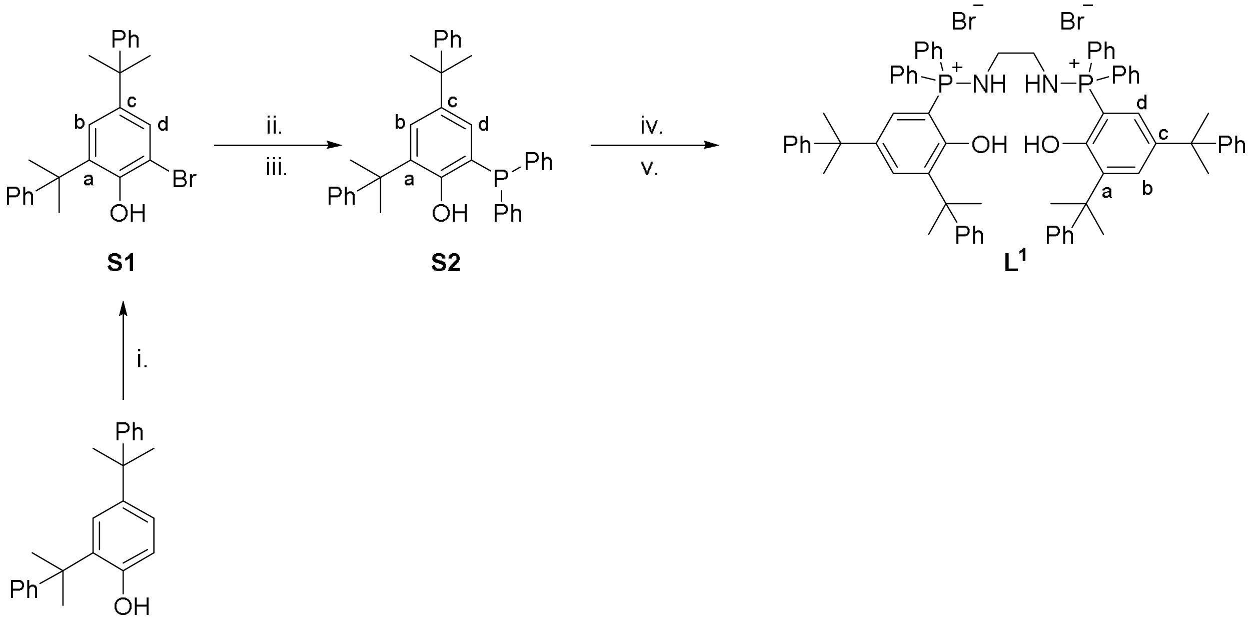

Scheme S1. i. NBS (1 eq.), acetonitrile **ii.** *n*-BuLi (2 eq.), Et_2O **iii.** $ClPPh_2$ (1 eq.) **iv.** Br_2 (1 eq.), CH_2Cl_2 **v.** Bu_3N (1 eq.), ethylene diamine (0.5 eq.).

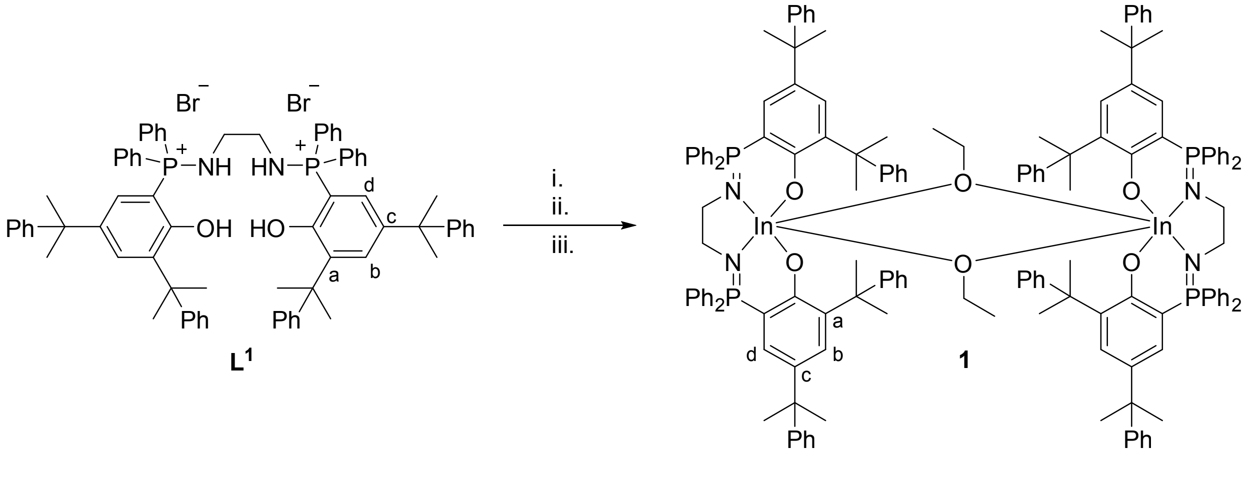

Scheme S2. i. NaH (6 eq.), THF **ii.** $InCl_3$ (1 eq.), **iii.** KOEt (1 eq.).

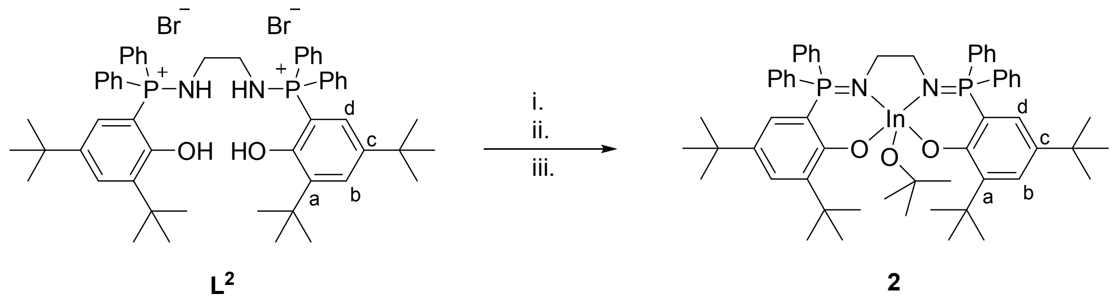

Scheme S3. i. NaH (6 eq.), THF **ii.** $InCl_3$ (1 eq.), **iii.** KO^tBu (1 eq.).

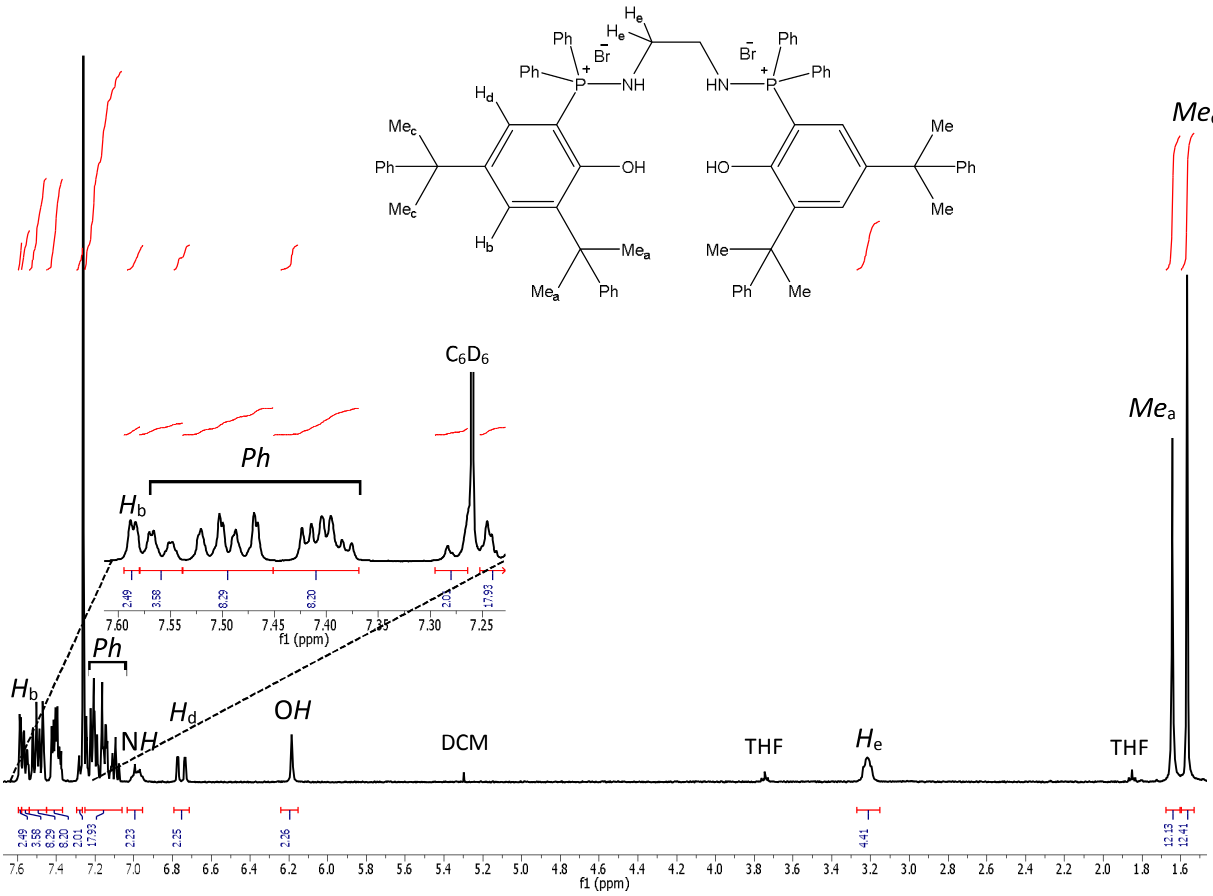

Figure S1. ^{1}H NMR spectrum of compound **L¹** in $CDCl_3$.

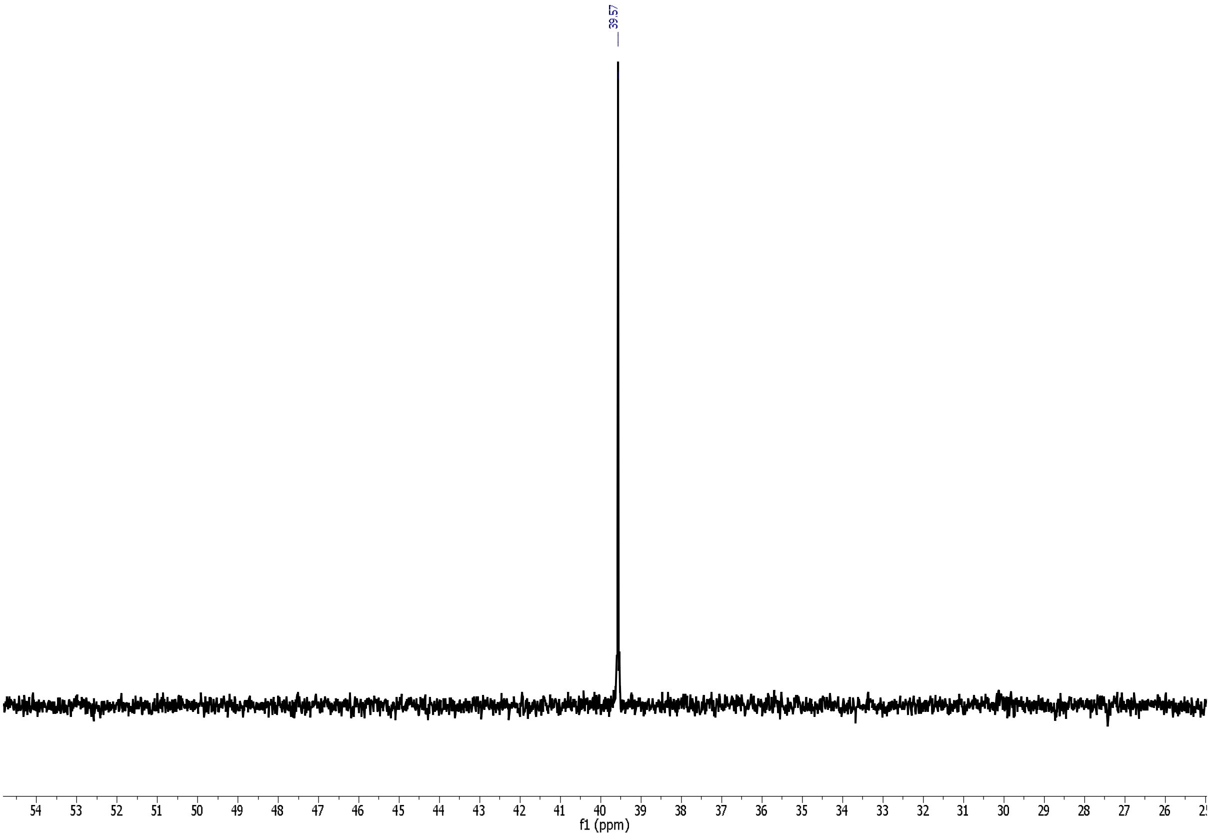

Figure S2. ^{31}P NMR spectrum of compound **L¹** in $CDCl_3$.

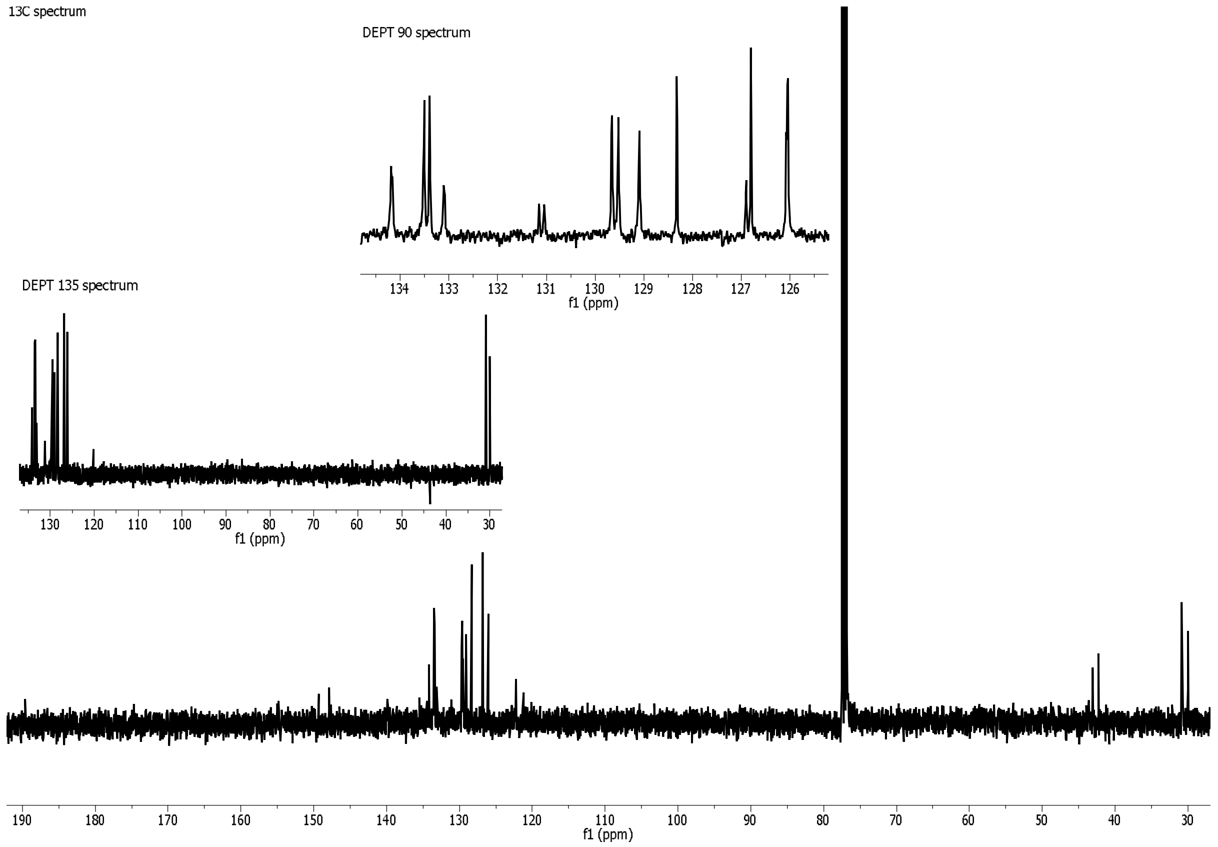

Figure S3. ^{13}C NMR spectrum of compound **L¹** in $CDCl_3$. (Bottom left inset) DEPT 135 spectrum. (Top right inset) DEPT 90 spectrum.

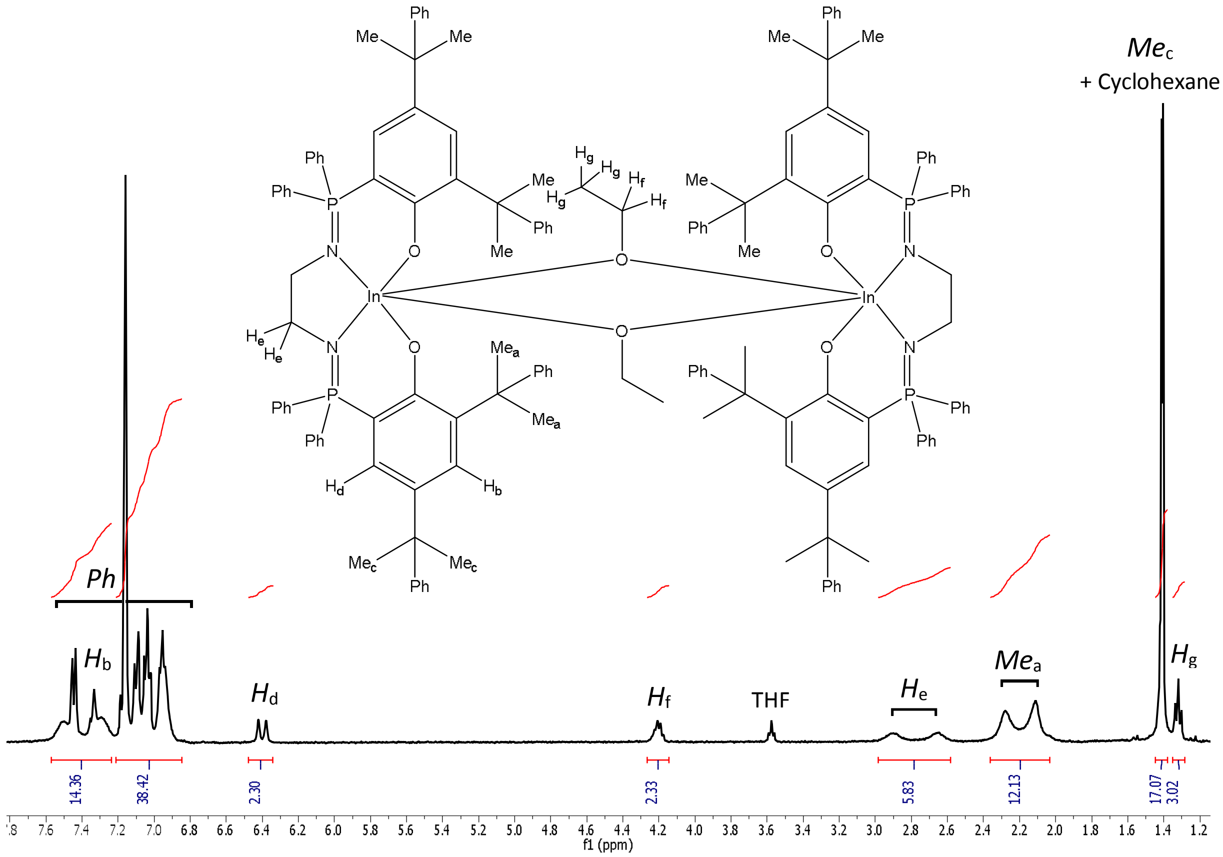

Figure S4. ^{1}H NMR spectrum of compound **1** in C_6D_6.

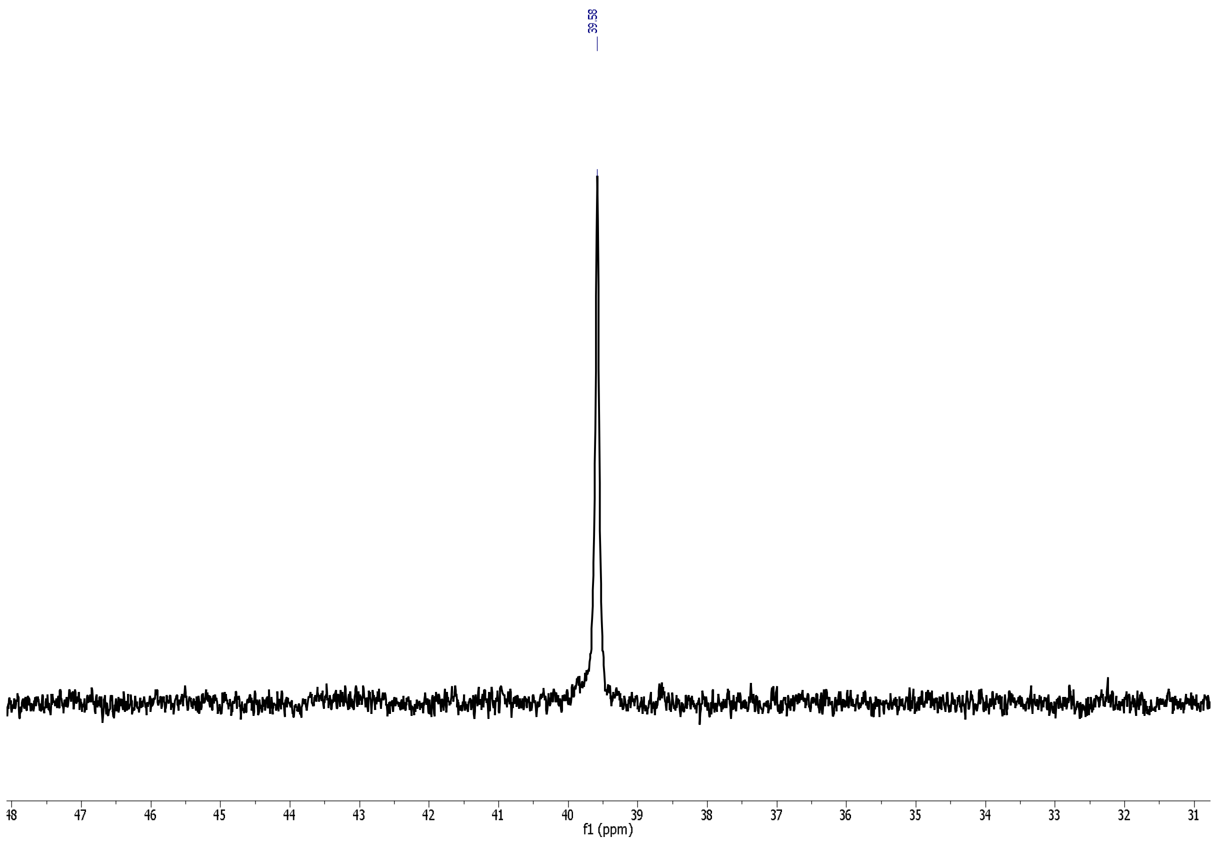

Figure S5. ^{31}P NMR spectrum of compound **1** in C_6D_6

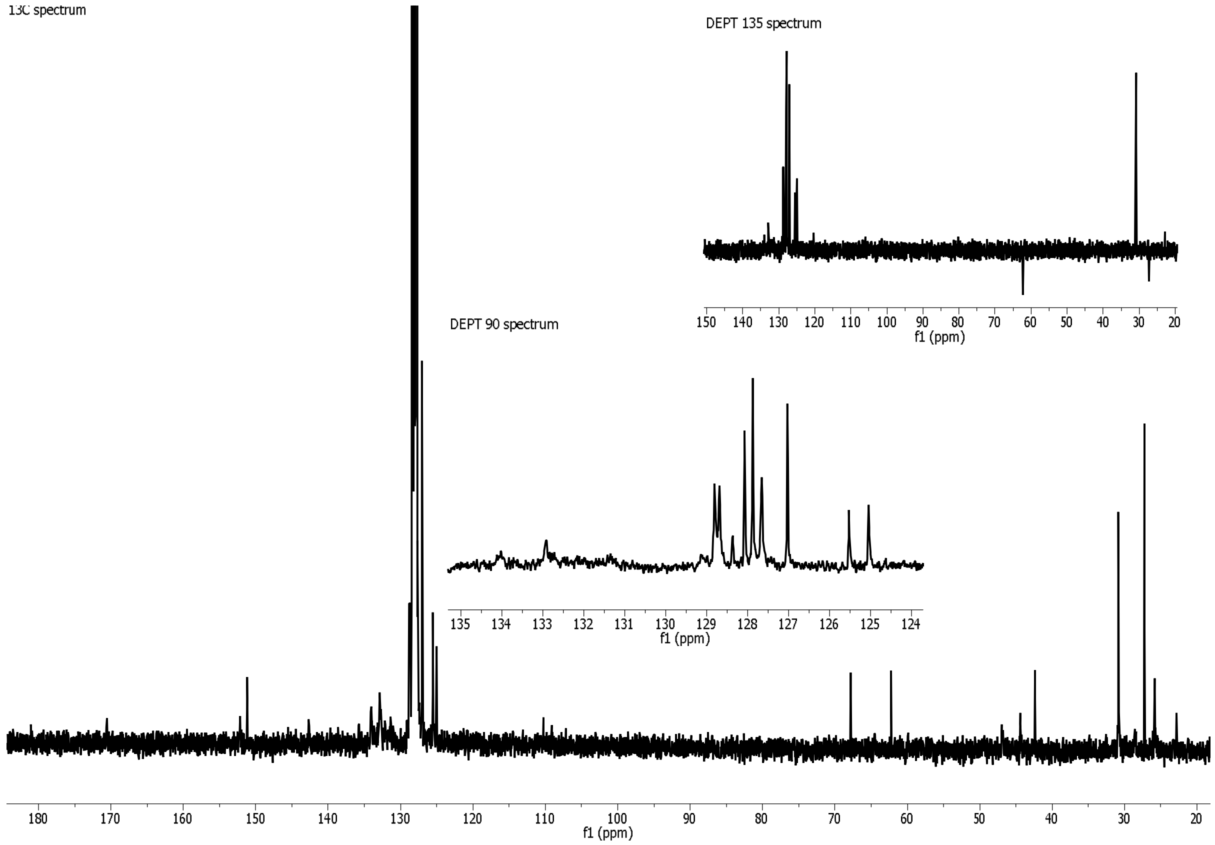

Figure S6. ^{13}C NMR spectrum of compound **1** in C_6D_6. (Bottom left inset) DEPT 90 spectrum. (Top right inset) DEPT 135 spectrum.

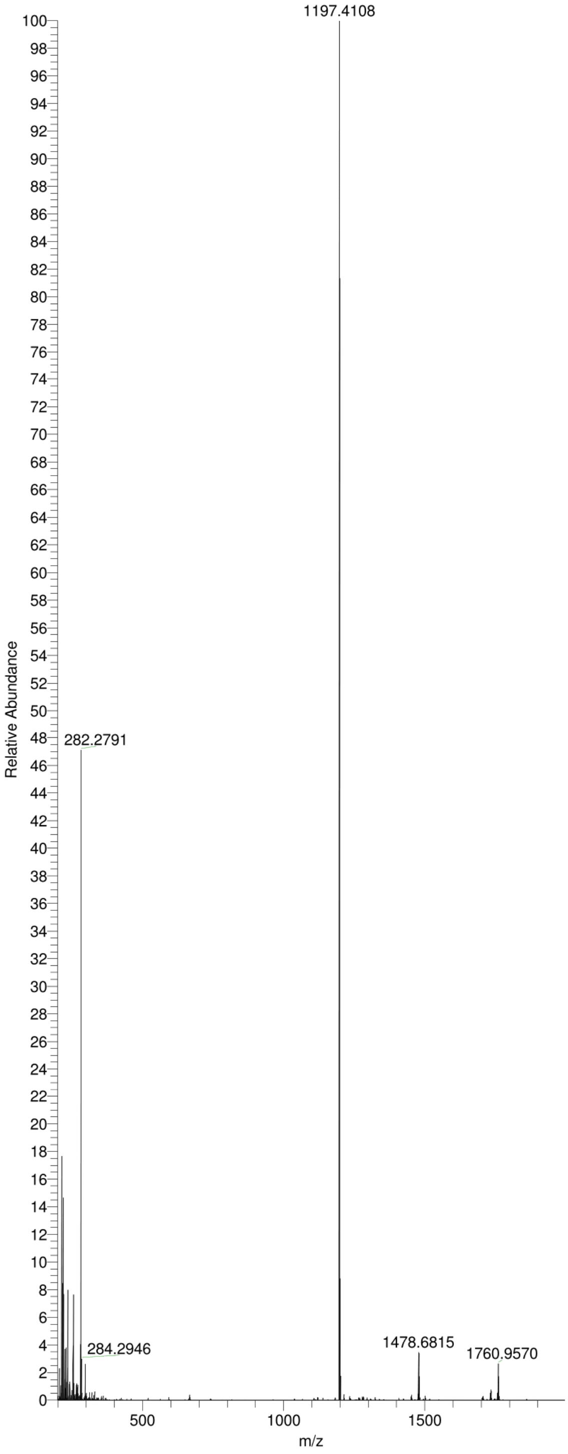

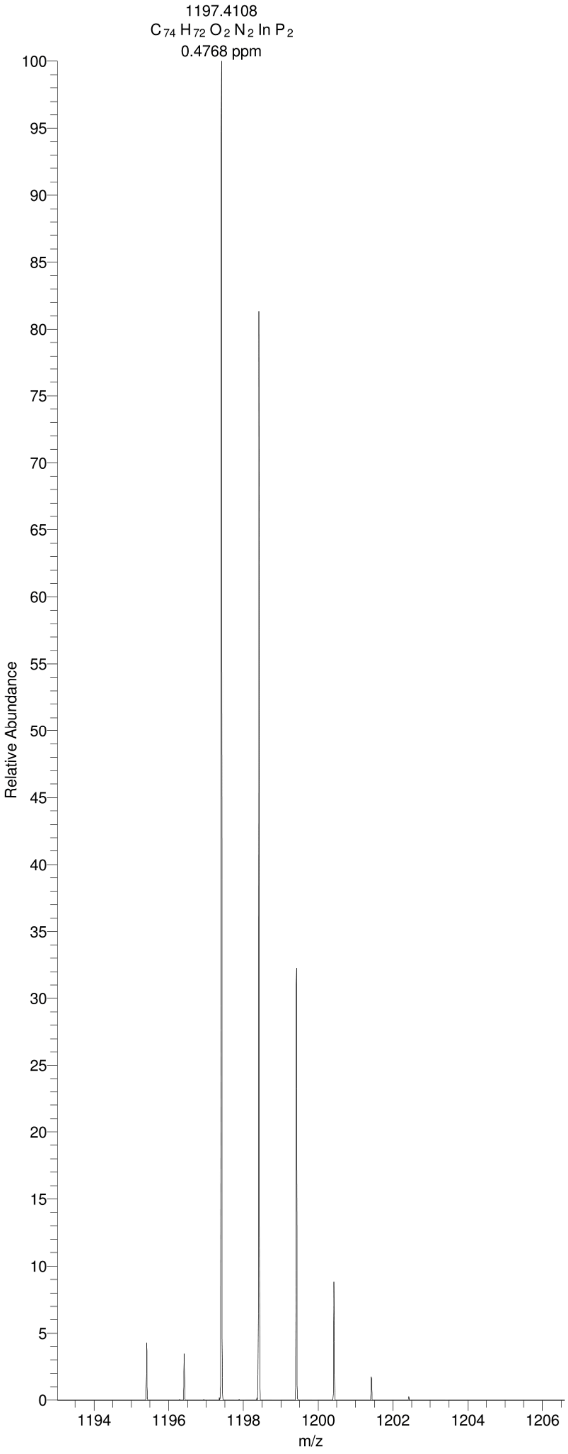

Figure S7. (Left) Electrospray (ES) ionisation mass spectrum (MS) of compound **1**. (Right) Close-up zoom of the molecular ion peak in the ES-MS of compound **1**. The molecular ion peak is identified as $[M\text{-}OEt]^{+\cdot}$

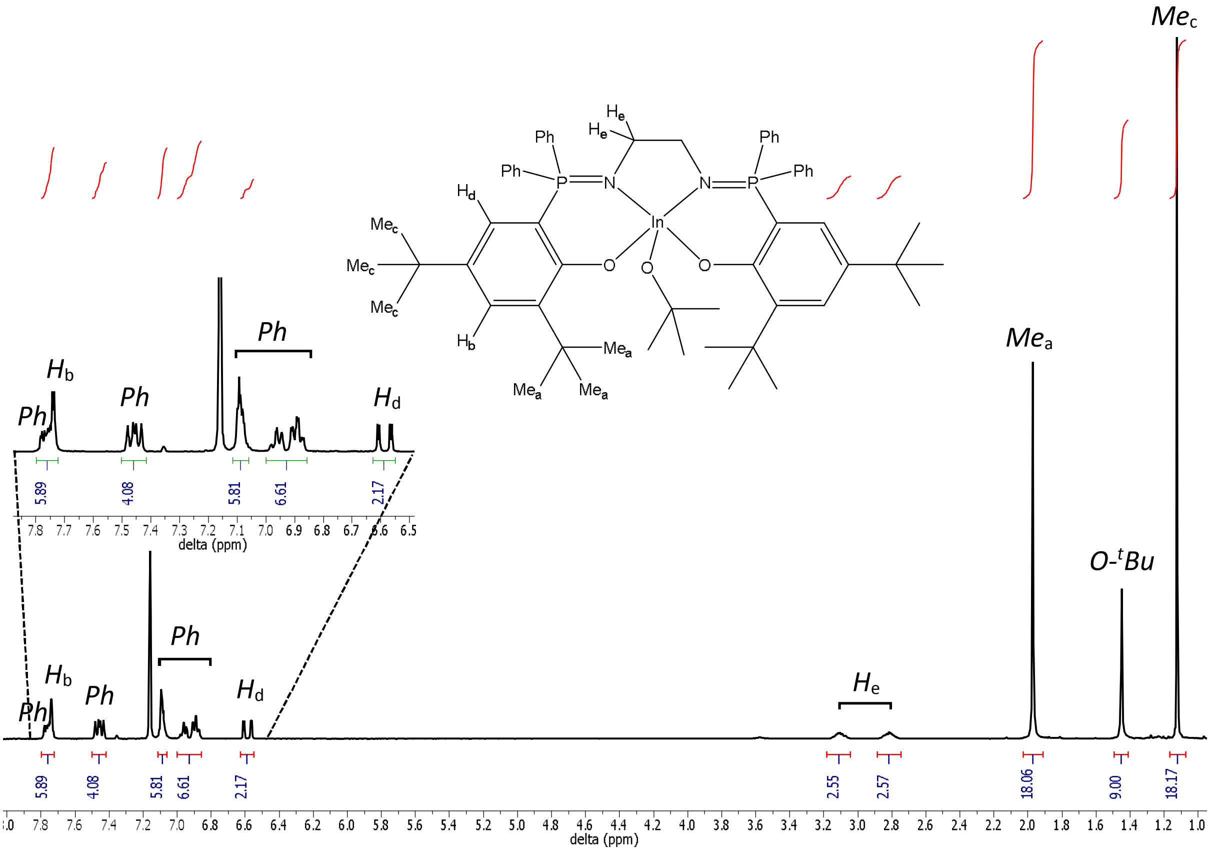

Figure S8. ^{1}H NMR spectrum of compound **2** in C_6D_6

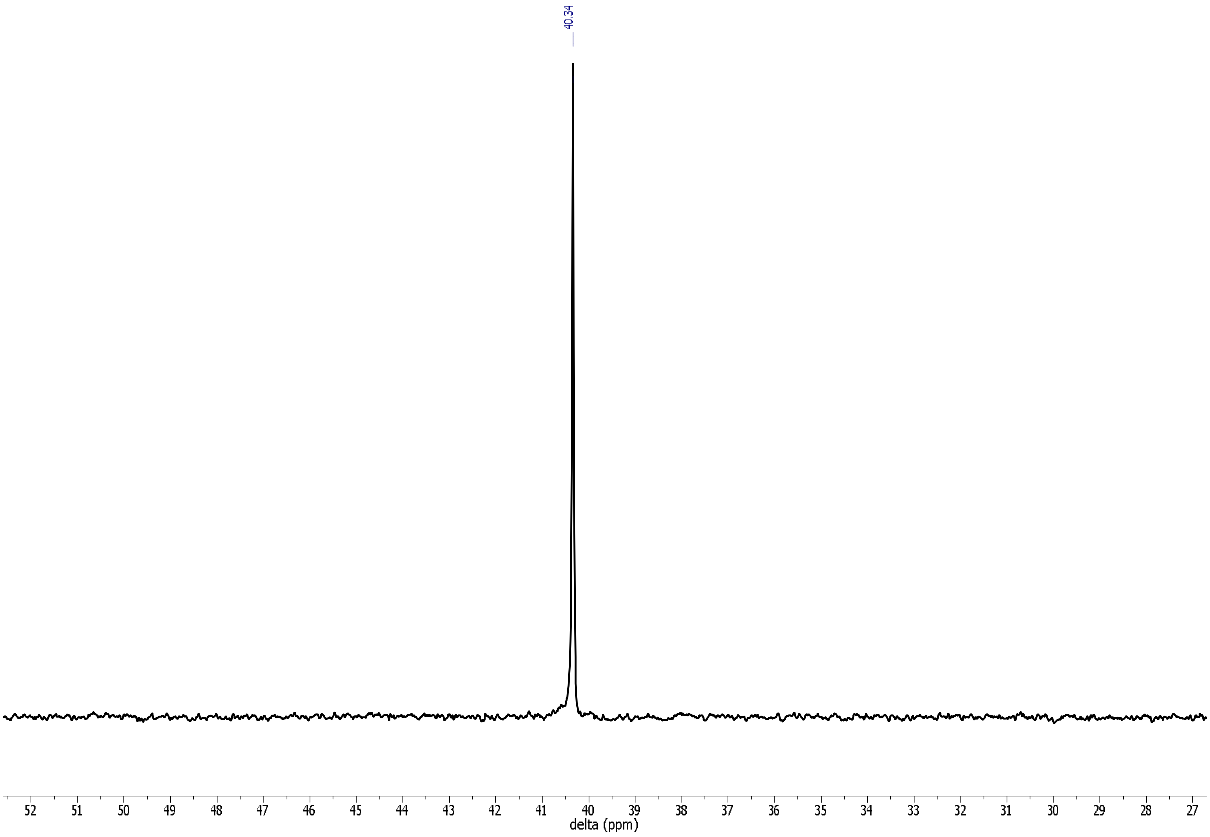

Figure S9. ^{31}P NMR spectrum of compound **2** in C_6D_6

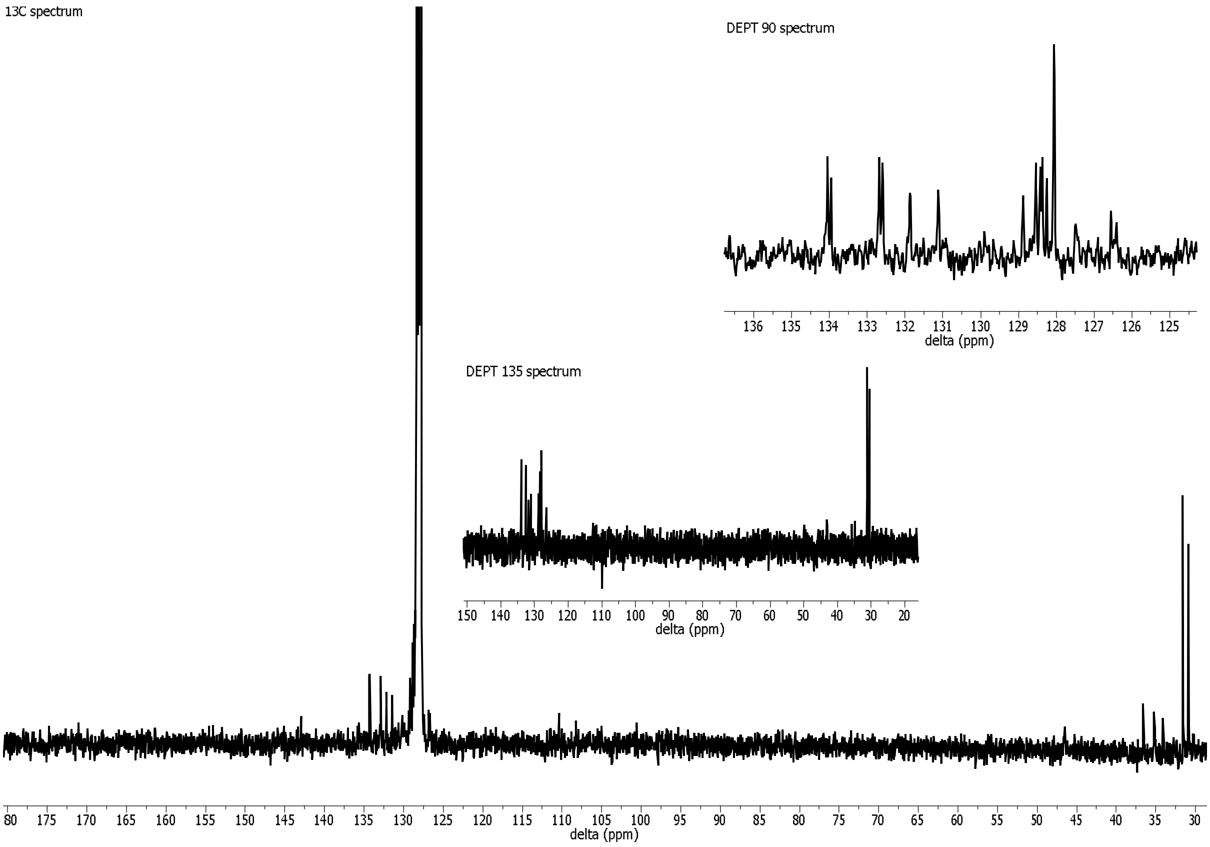

Figure S10. ^{13}C NMR spectrum of compound **2** in C_6D_6. (Bottom left inset) DEPT 135 spectrum. (Top right inset) DEPT 90 spectrum.

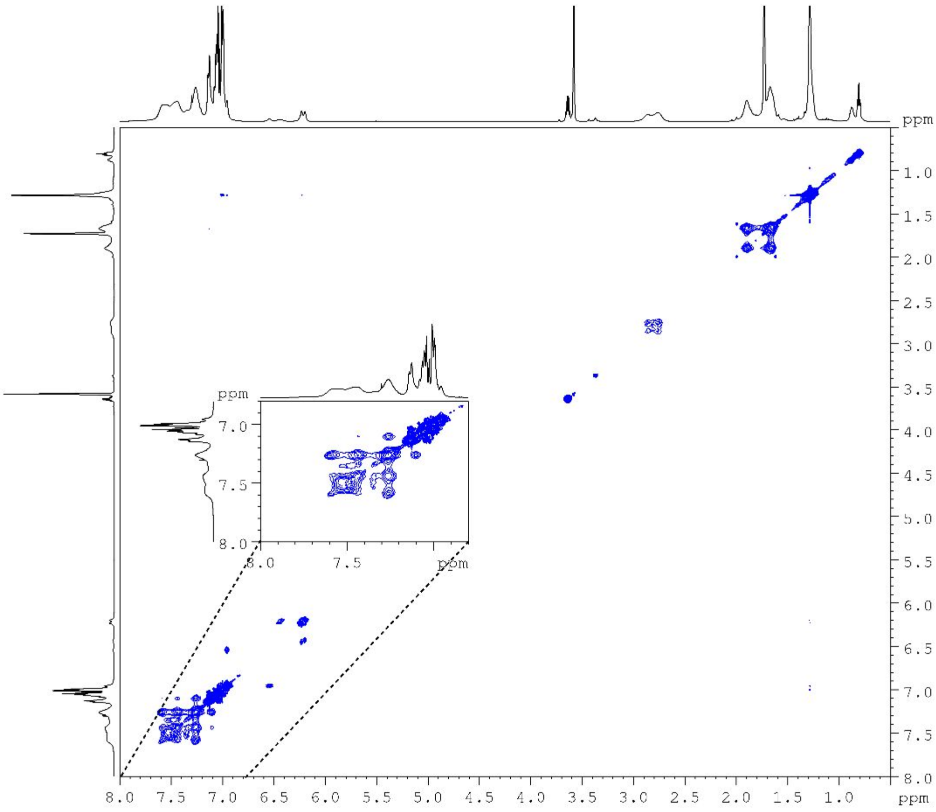

Figure S11. ^{1}H ROESY NMR spectrum of compound **1** in THF-d_8

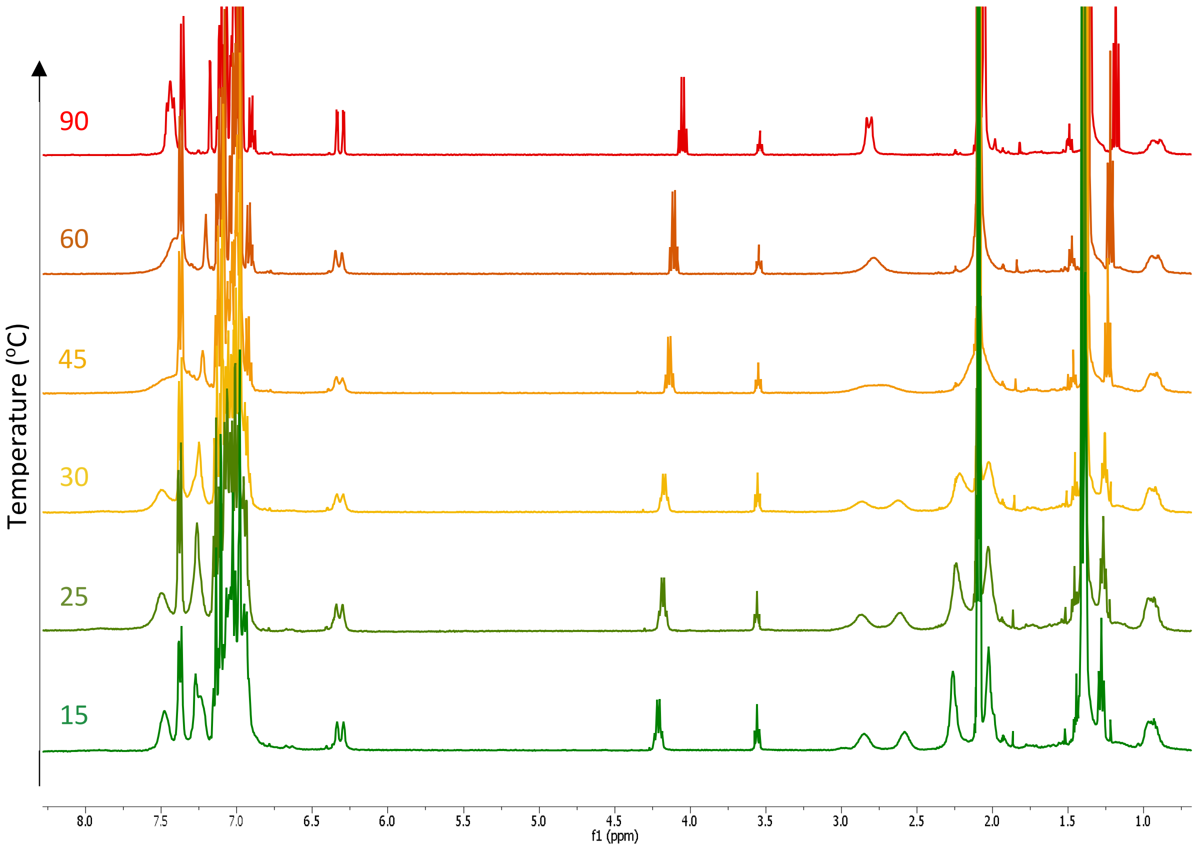

Figure S12. ^{1}H VT NMR spectrum of compound **1** in toluene-d_8

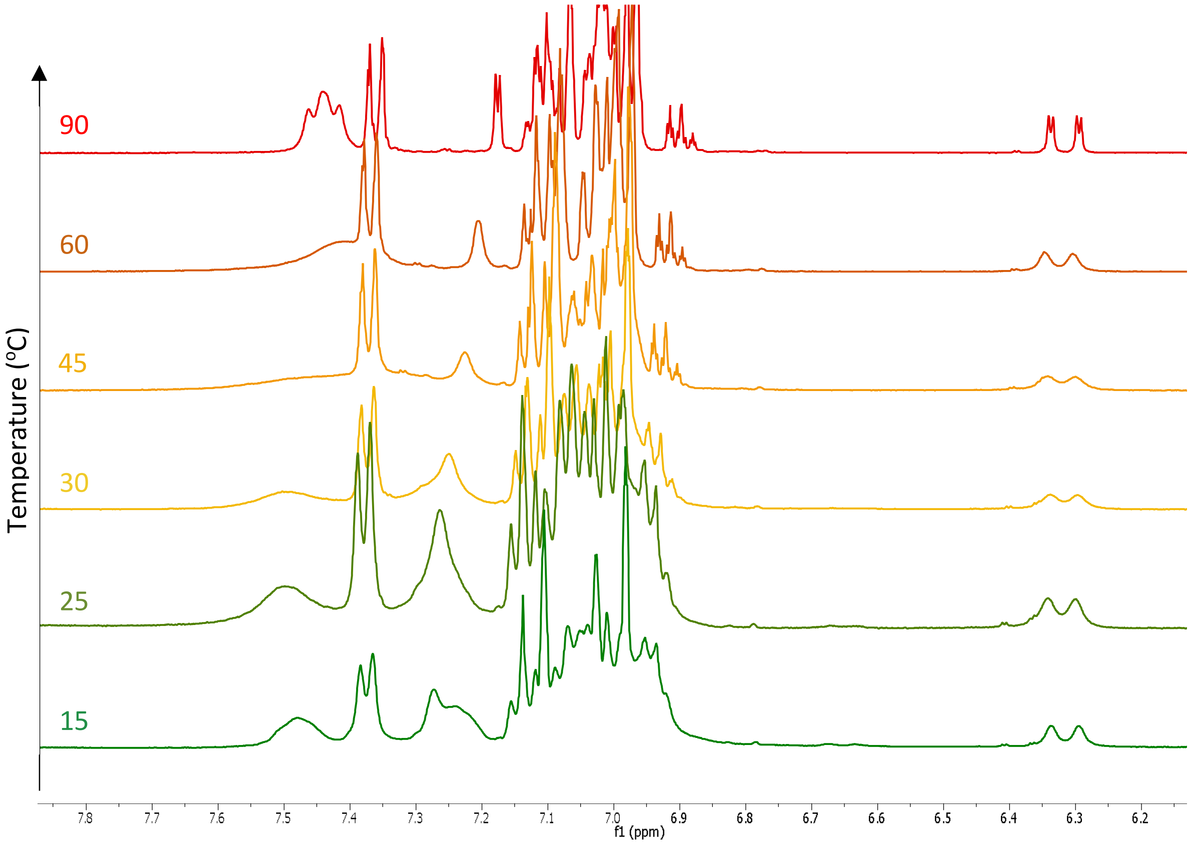

Figure S13. ^{1}H VT NMR spectrum of compound **1** (zoom on aromatic resonances) in toluene-d_8

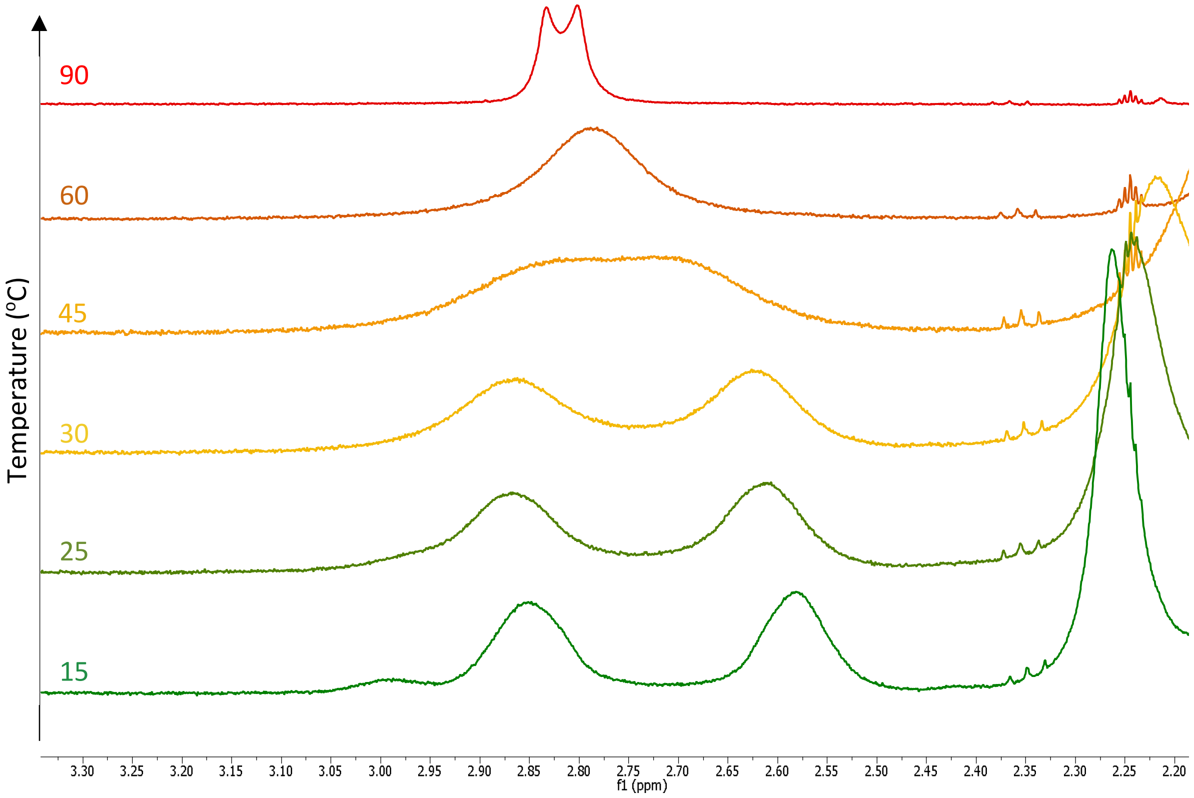

Figure S14. ^{1}H VT NMR spectrum of compound **1** (zoom on ethylene backbone resonances) in toluene-d_8

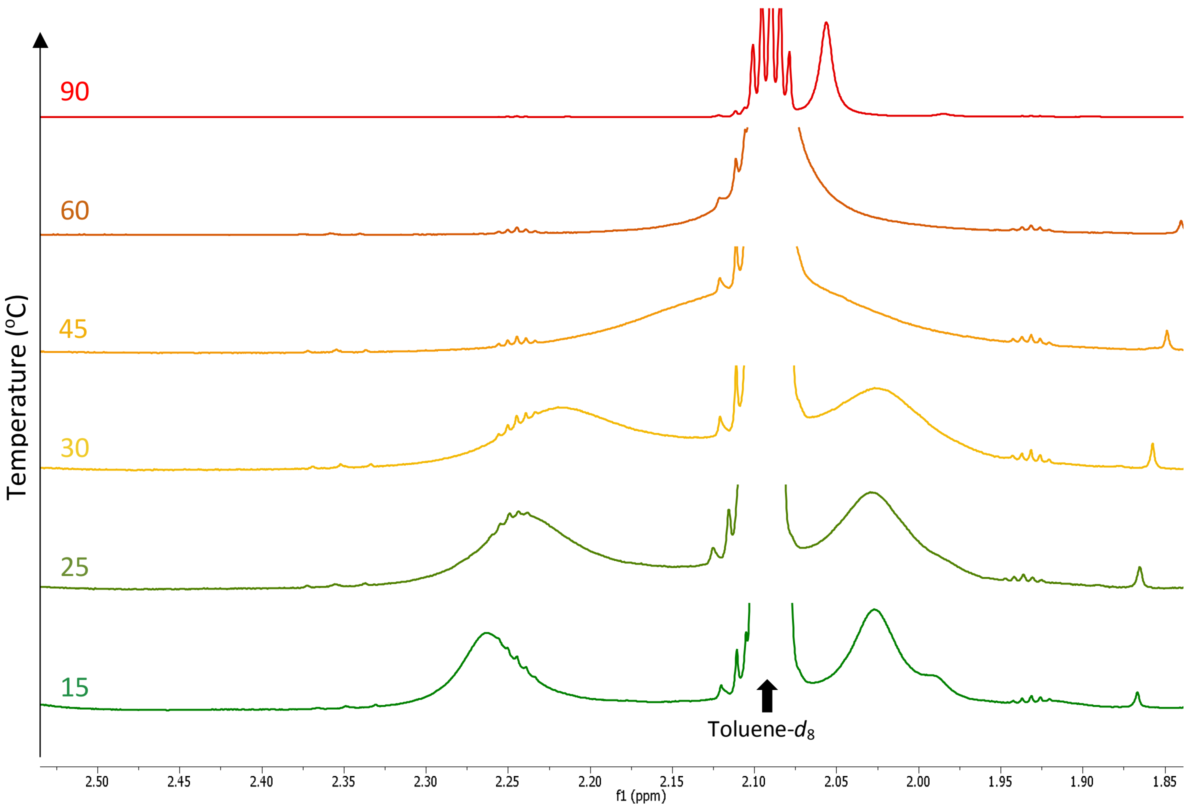

Figure S15. ^{1}H VT NMR spectrum of compound **1** (zoom on *ortho*-cumyl methyl group resonances) in toluene-d_8

The X-ray crystal structure of 1

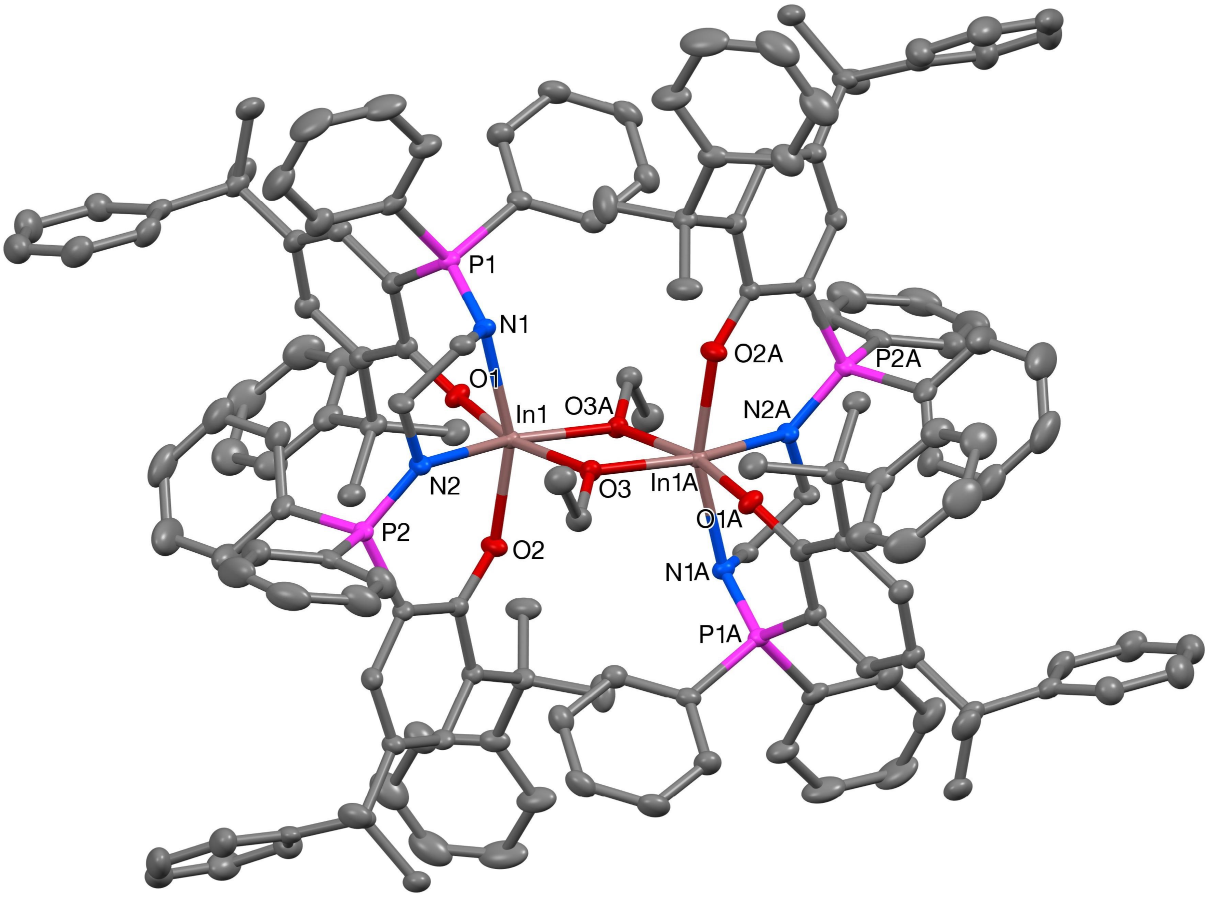

Figure S16. The crystal structure of the C_i-symmetric complex **1** (50% probability ellipsoids).

Crystal data for **1**: $C_{152}H_{154}In_2N_4O_6P_4{\cdot}2(C_4H_8O)$, M = 2630.68, triclinic, P-1 (no. 2), a = 12.4939(5), b = 14.4659(7), c = 18.9309(9) Å, α = 102.475(2), β = 91.358(2), γ = 103.240(2)°, V = 3242.3(3) Å^3, Z = 1 [C_i symmetry], ρ_{calcd} = 1.347 g cm^{-3}, $\mu(Mo_{K\alpha})$ = 0.468 mm^{-1}, T = 123 K, colourless plates, Bruker APEX II CCD diffractometer; 15649 independent measured reflections (R_{int} = 0.0418), F^2 refinement,[5] R_1(obs) = 0.0318, wR_2(all) = 0.0767, 13380 independent observed absorption-corrected reflections [$|F_o| > 4\sigma(|F_o|)$, $2\theta_{full}$ = 50°], 802 parameters. CCDC 1523645.

The data were collected and processed, including an empirical absorption correction, using the proprietary software Apex2 v2014.1-7.[5] The structure was solved and refined by conventional methods using the SHELX-2014 software package.[6] Non-hydrogen atoms were refined with anisotropic displacement parameters and hydrogen atoms were placed in calculated positions using a riding model. One of the substituent phenyl rings C(69)-C(74) exhibited some unusual anisotropic displacement parameters plausibly indicative of disorder. However this could not be modelled with the current data.

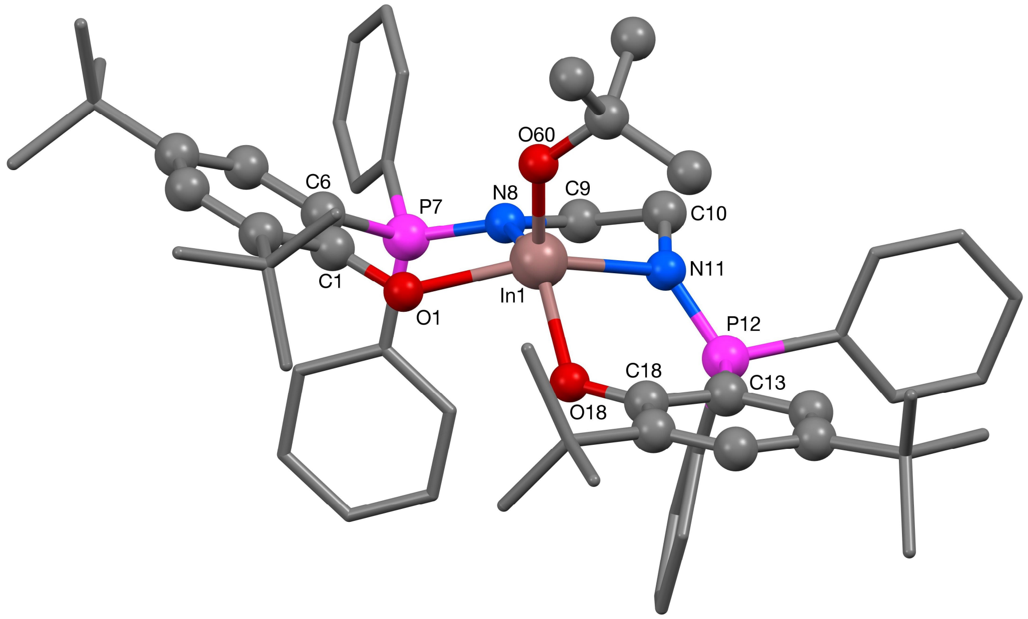

Figure S17. The crystal structure of **2**.

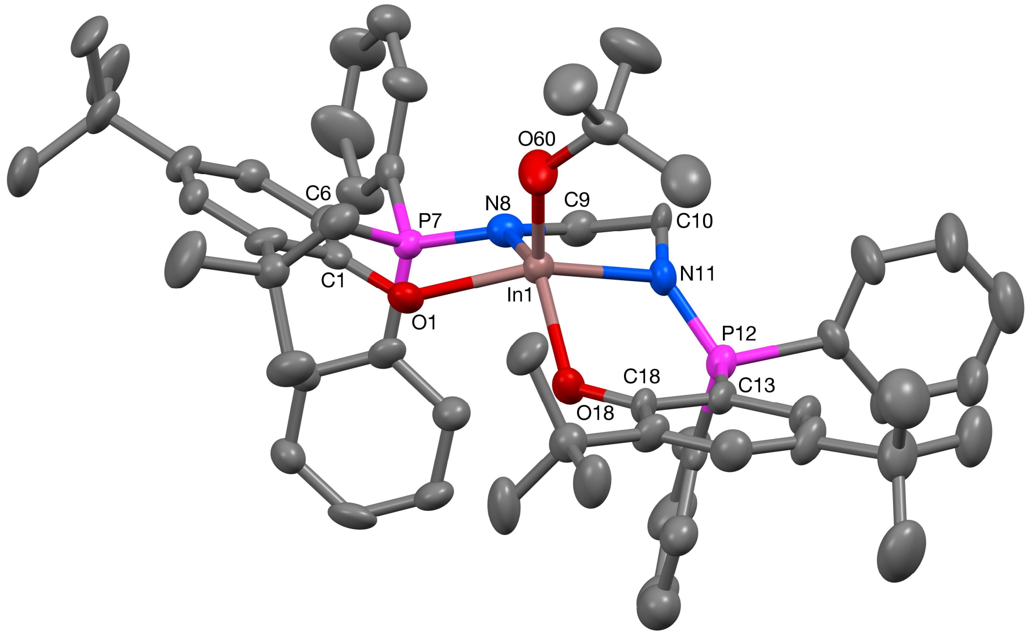

Figure S18. The crystal structure of **2** (50% probability ellipsoids).

Crystal data for **2**: $C_{58}H_{73}InN_2O_3P_2 \cdot 5.5(C_4H_8O)$, M = 1419.51, monoclinic, $P2_1/c$ (no. 14), a = 15.5751(6), b = 17.6361(9), c = 29.0862(14) Å, β = 96.324(4)°, V = 7940.9(6) Å^3, Z = 4, ρ_{calcd} = 1.187 g cm^{-3}, $\mu(Mo_{K\alpha})$ = 0.391 mm^{-1}, T = 173 K, colourless needles, Agilent Xcalibur 3 E diffractometer; 15695 independent measured reflections (R_{int} = 0.0449), F^2 refinement,[6-7] R_1(obs) = 0.1209, wR_2(all) = 0.3071, 13049 independent observed absorption-corrected reflections [$|F_o| > 4\sigma(|F_o|)$, $2\theta_{full}$ = 50°], 639 parameters. CCDC 1523646.

Reciprocal space analysis of the data set for the structure of **2** clearly showed the crystal to be twinned. Despite numerous attempts, modelling this twinning at the data processing stage gave unsatisfactory results, and so the data was processed without any twin modelling.

The included solvent was found to be highly disordered, and the best approach to handling this diffuse electron density was found to be the SQUEEZE routine of PLATON.[8] This suggested a total of 905 electrons per unit cell, equivalent to 226.25 electrons per asymmetric unit. Before the use of SQUEEZE the solvent clearly resembled thf (C_4H_8O, 40 electrons), and 5.5 thf molecules corresponds to 220 electrons, so this was used as the solvent present. As a result, the atom list for the asymmetric unit is low by 5.5(C_4H_8O) = $C_{22}H_{44}O_{5.5}$ (and that for the unit cell low by $C_{88}H_{176}O_{22}$) compared to what is actually presumed to be present.

The C23- and C51-based *t*-butyl groups were found to be disordered, and in each case two orientations were identified, of *ca*. 79:21 and 85:15% occupancy respectively. The geometries of each pair of orientations were optimised, the thermal parameters of adjacent atoms were restrained to be similar, and only the non-hydrogen atoms of the major occupancy orientations were refined anisotropically (those of the minor occupancy orientations were refined isotropically).

Table S1. Selected bond lengths (Å) and angles (°) for compound **1**.

In(1) – O(3)	2.1213(12)	O(3)-In(1)-O(1)	92.57(5)	O(1)-In(1)-N(1)	84.32(5)
In(1) – O(2)	2.1328(13)	O(2)-In(1)-O(1)	86.64(5)	N(2)-In(1)-N(1)	77.55(5)
In(1) – O(1)	2.1781(13)	O(3)-In(1)-N(2)	161.93(6)	O(3)-In(1)-O(3)i	73.18(5)
In(1) – N(2	2.1921(15)	O(2)-In(1)-N(2)	87.91(5)	O(2)-In(1)-O(3)i	98.47(5)
In(1) – N(1)	2.2379(15)	O(1)-In(1)-N(2)	105.49(6)	O(1)-In(1)-O(3A)	165.07(5)
In(1) – O(3A)	2.2385(13)	O(3)-In(1)-N(1)	104.32(5)	N(2)-In(1)-O(3A)	88.78(5)
O(3)-In(1)-O(2)	93.72(5)	O(2)-In(1)-N(1)	160.12(5)	N(1)-In(1)-O(3A)	94.74 (5)

Table S2. Selected bond lengths (Å) and angles (°) for compound **2**.

In(1) – O(60)	2.023(5)	O(60)-In(1)-O(18)	110.0(2)	O(1)-In(1)-N(8)	87.28(17)
In(1) – O(18)	2.112(4)	O(60)-In(1)-O(1)	94.04(18)	O(60)-In(1)-N(11)	112.4(2)
In(1) – O(1)	2.122(4)	O(18)-In(1)-O(1)	87.87(16)	O(18)-In(1)-N(11)	87.08(18)
In(1) – N(8)	2.187(5)	O(60)-In(1)-N(8)	116.6(2)	O(1)-In(1)-N(11)	153.20(18)
In(1) – N(11)	2.203(5)	O(18)-In(1)-N(8)	133.40(18)	N(8)-In(1)-N(11)	77.30(19)

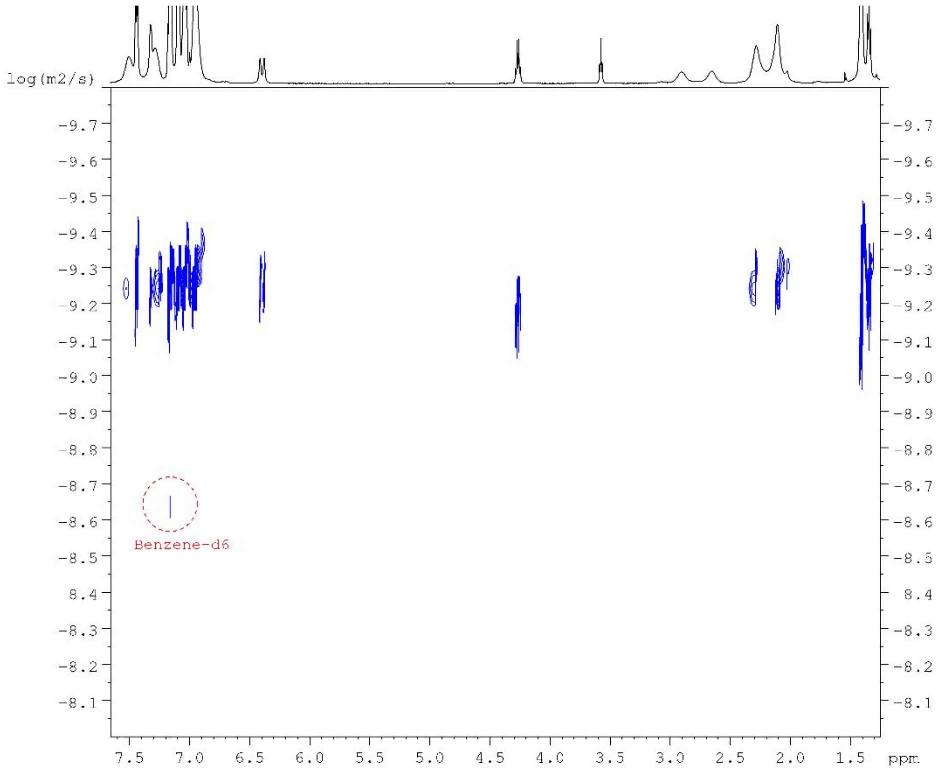

Figure S19. ^{1}H DOSY NMR of compound **1** in benzene-d_6 at 298 K

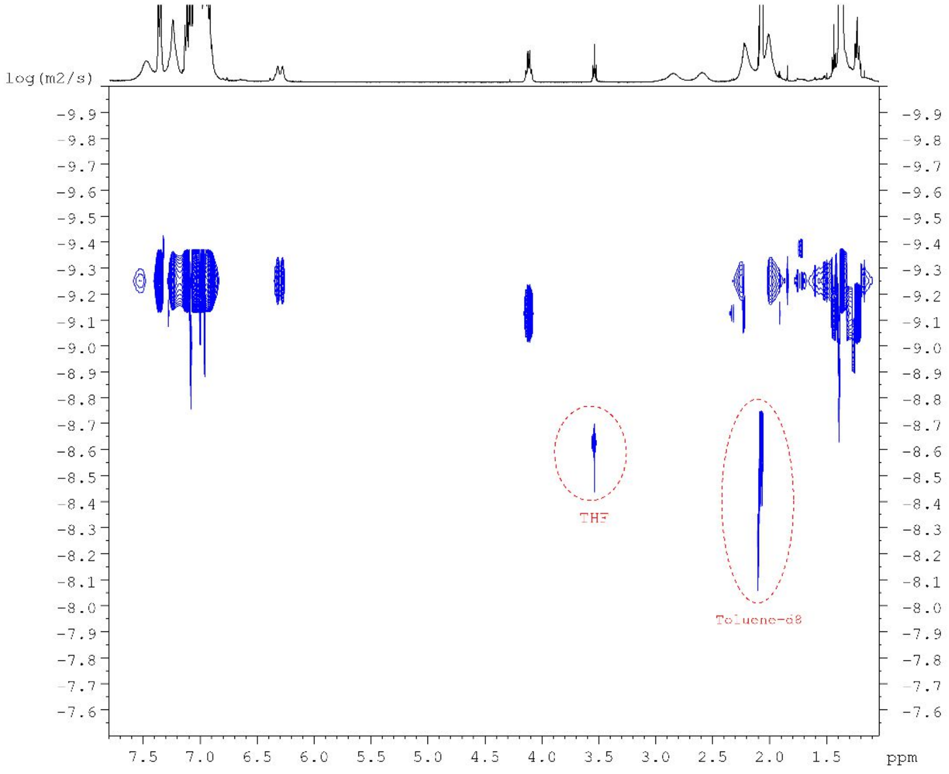

Figure S20. ^{1}H DOSY NMR of compound **1** in toluene-d_8 at 298 K

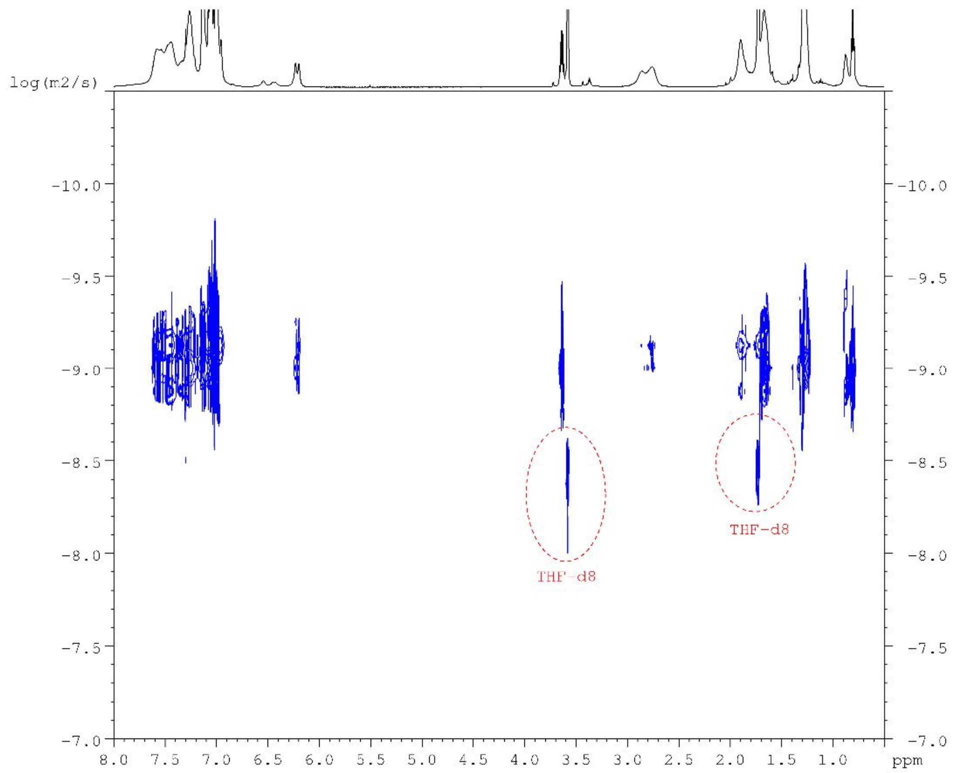

Figure S21. ^{1}H DOSY NMR of compound **1** in THF-d_8 at 298 K

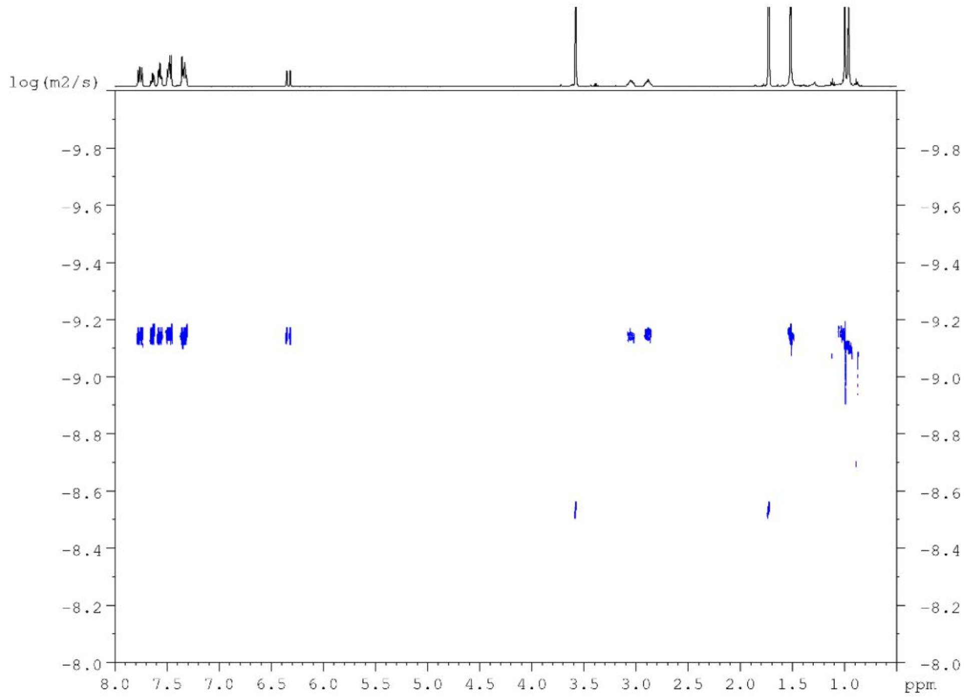

Figure S22. ^{1}H DOSY NMR of compound **2** in THF-d_8 at 298 K

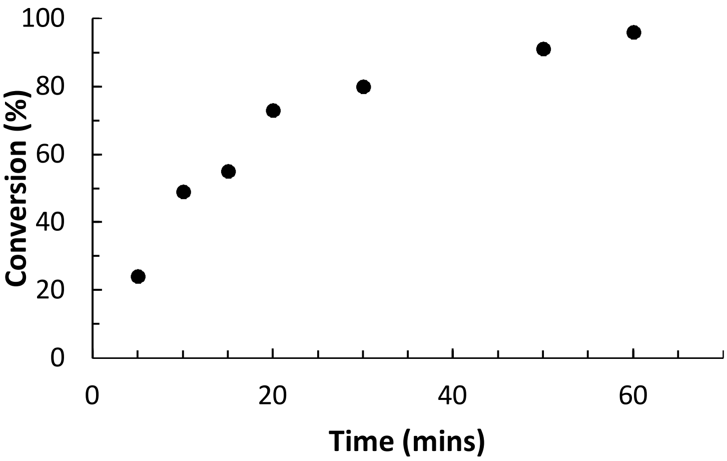

Figure S23. Conversion vs time plot for the polymerisation of *rac*-LA mediated by compound **1**. Conditions: [LA]/[**1**] = 500, [LA] = 1 M, THF, 298 K.

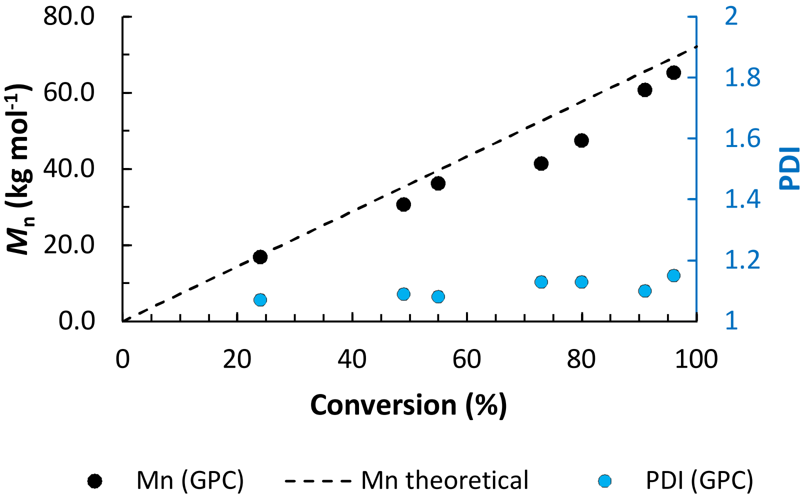

Figure S24. Plot of the molecular weight and polydispersity vs conversion for the polymerisation of *rac*-LA mediated by compound **1**. Conditions: [LA]/[**1**] = 500, [LA] = 1 M, THF, 298 K.

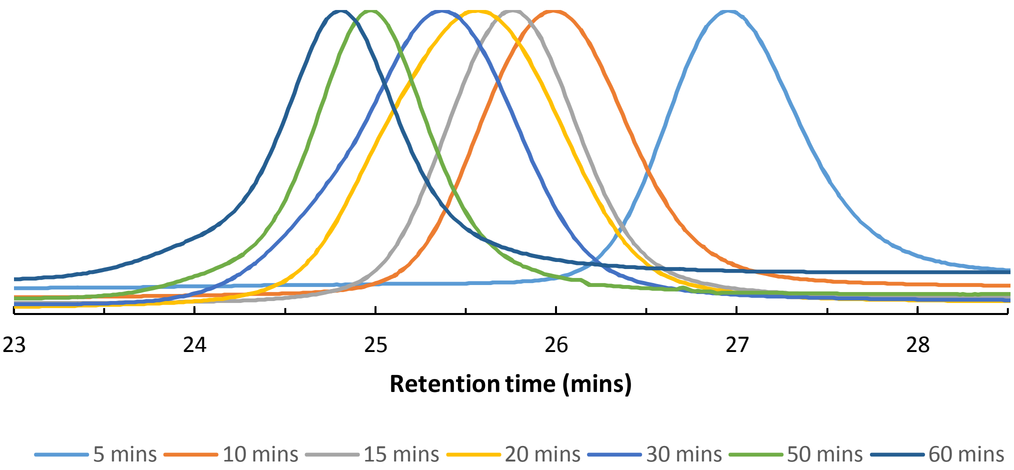

Figure S25. Overlay of SEC traces for the polymerisation of *rac*-LA mediated by **1**. Conditions: [LA]/[**1**] = 500, [LA] = 1 M, THF, 298 K

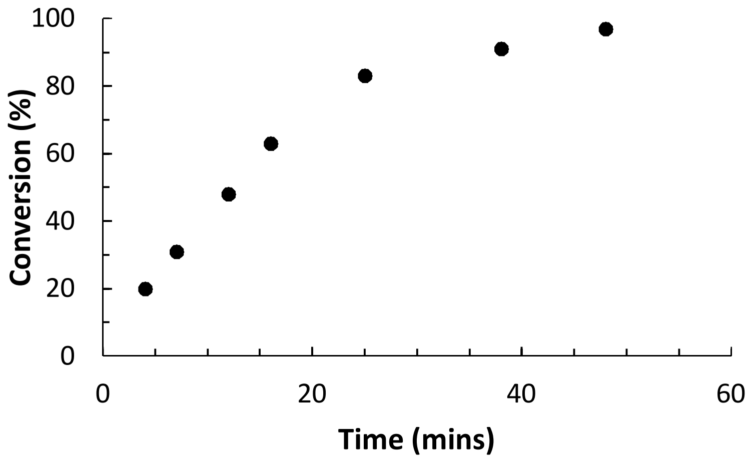

Figure S26. Conversion vs time plot for the polymerisation of *rac*-LA mediated by compound **1**. Conditions: [LA]/[**1**] = 400, [LA] = 1 M, THF, 298 K.

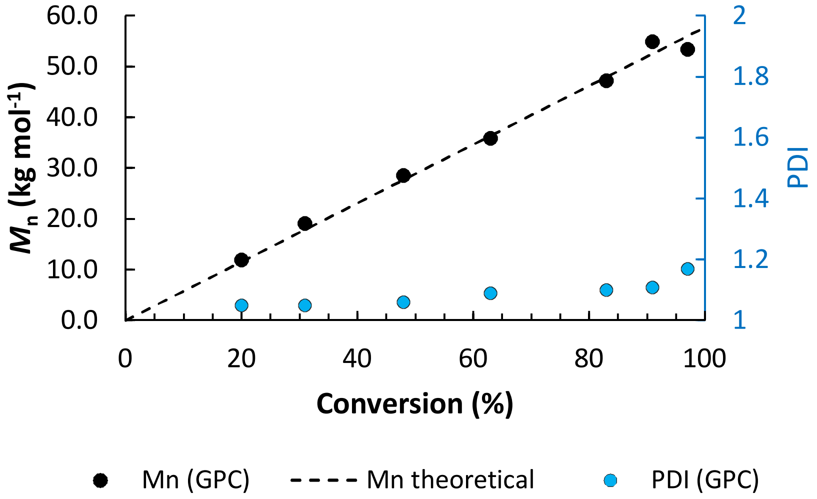

Figure S27. Plot of the molecular weight and polydispersity vs conversion for the polymerisation of *rac*-LA mediated by compound **1**. Conditions: [LA]/[**1**] = 400, [LA] = 1 M, THF, 298 K.

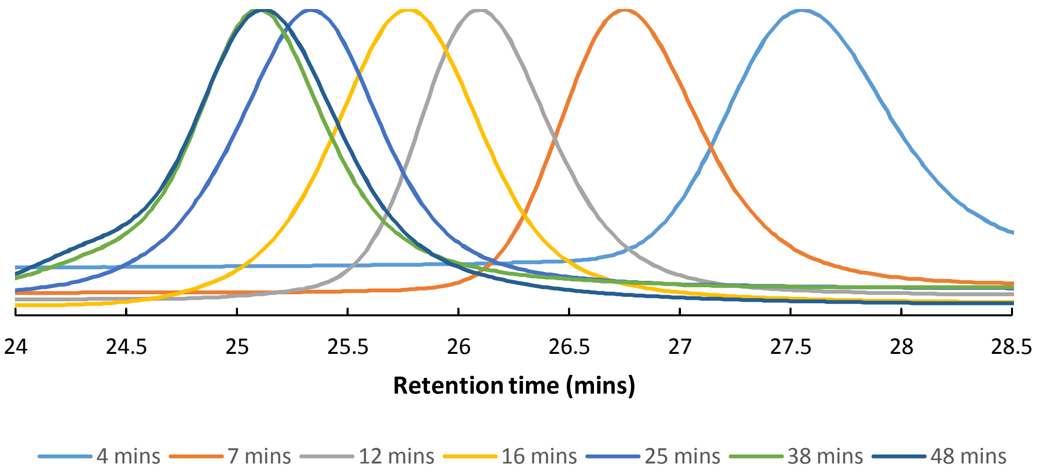

Figure S28. Overlay of SEC traces for the polymerisation of *rac*-LA mediated by **1**. Conditions: [LA]/[**1**] = 400, [LA] = 1 M, THF, 298 K.

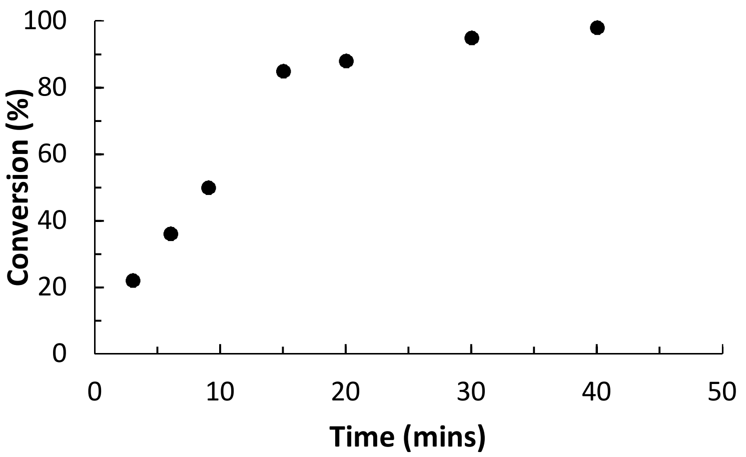

Figure S29. Conversion vs time plot for the polymerisation of *rac*-LA mediated by compound **1**. Conditions: [LA]/[**1**] = 300, [LA] = 1 M, THF, 298 K.

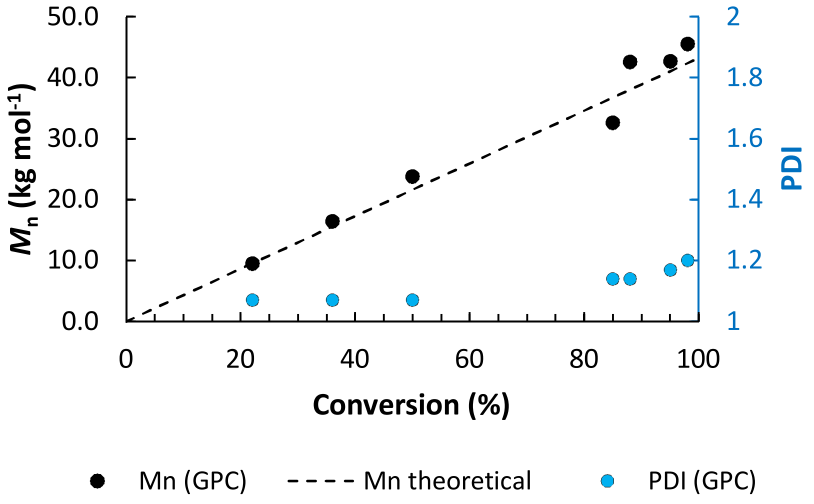

Figure S30. Plot of the molecular weight and polydispersity vs conversion for the polymerisation of *rac*-LA mediated by compound **1**. Conditions: [LA]/[**1**] = 300, [LA] = 1 M, THF, 298 K.

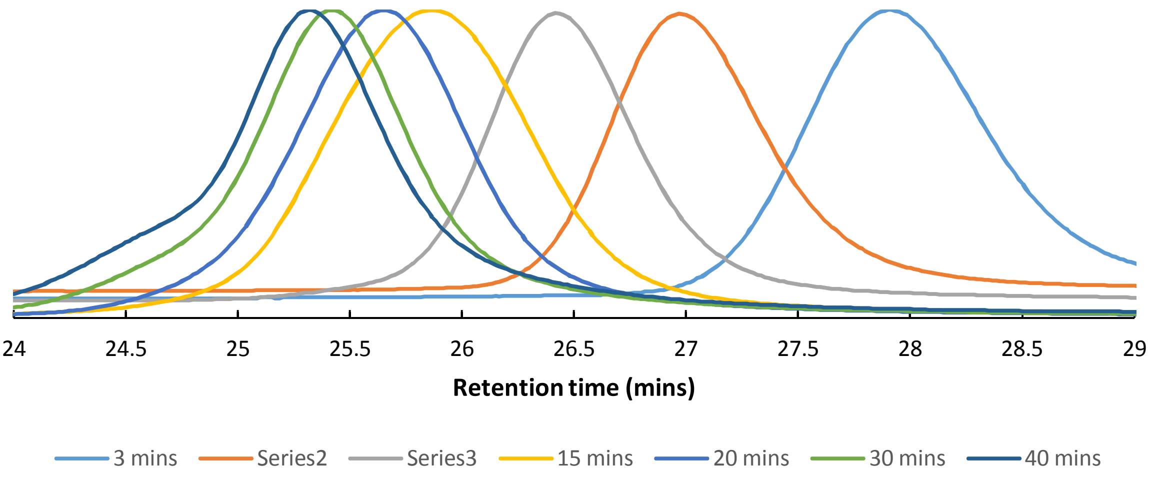

Figure S31. Overlay of SEC traces for the polymerisation of *rac*-LA mediated by **1**. Conditions: [LA]/[**1**] = 300, [LA] = 1 M, THF, 298 K.

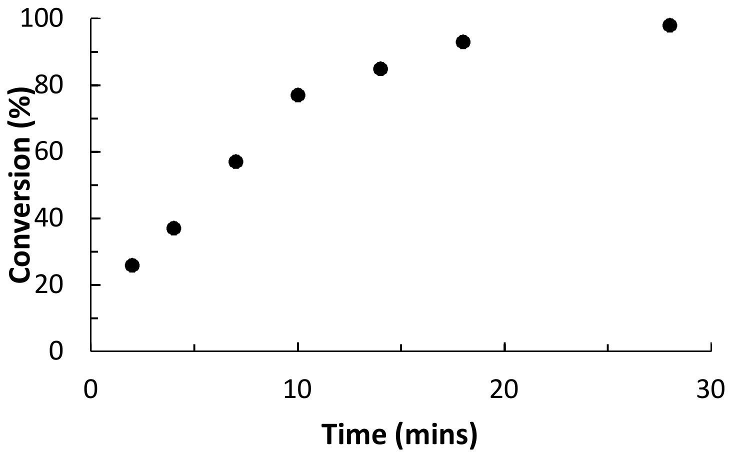

Figure S32. Conversion vs time plot for the polymerisation of *rac*-LA mediated by compound **1**. Conditions: [LA]/[**1**] = 200, [LA] = 1 M, THF, 298 K.

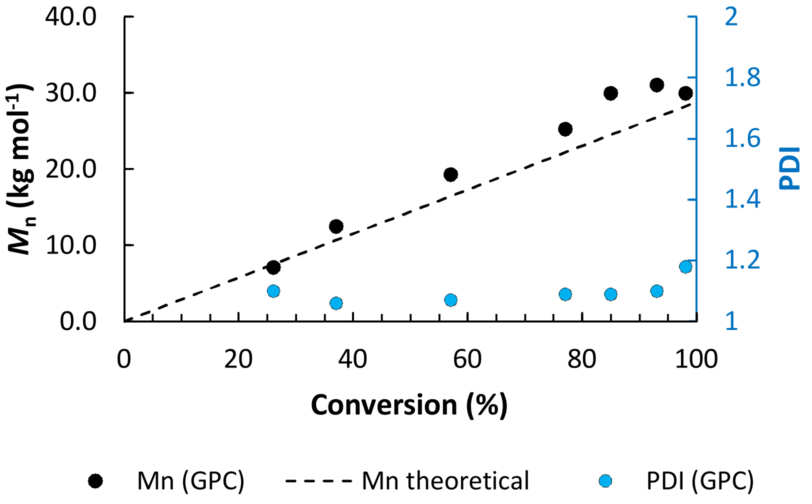

Figure S33. Plot of the molecular weight and polydispersity vs conversion for the polymerisation of *rac*-LA mediated by compound **1**. Conditions: [LA]/[**1**] = 200, [LA] = 1 M, THF, 298 K.

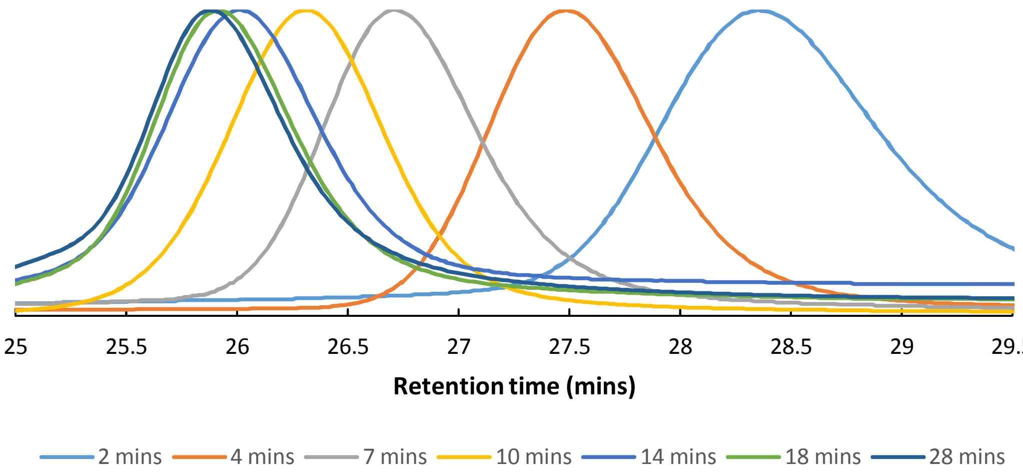

Figure S34. Overlay of SEC traces for the polymerisation of *rac*-LA mediated by **1**. Conditions: [LA]/[**1**] = 200, [LA] = 1 M, THF, 298 K.

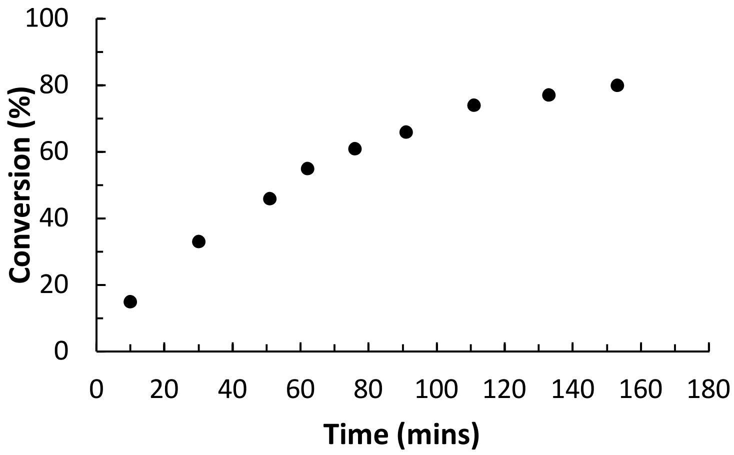

Figure S35. Conversion vs time plot for the polymerisation of *rac*-LA mediated by compound **2**. Conditions: [LA]/[**2**] = 500, [LA] = 1 M, THF, 298 K.

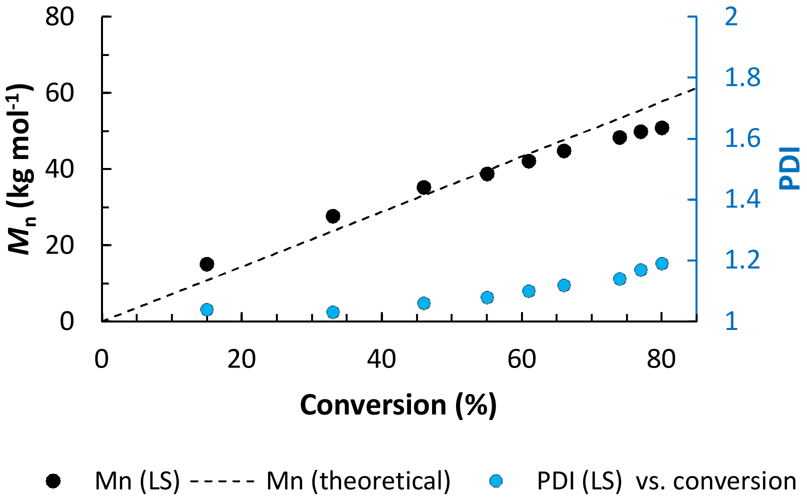

Figure S36. Plot of the molecular weight and polydispersity vs conversion for the polymerisation of *rac*-LA mediated by compound **2**. Conditions: [LA]/[**2**] = 500, [LA] = 1 M, THF, 298 K.

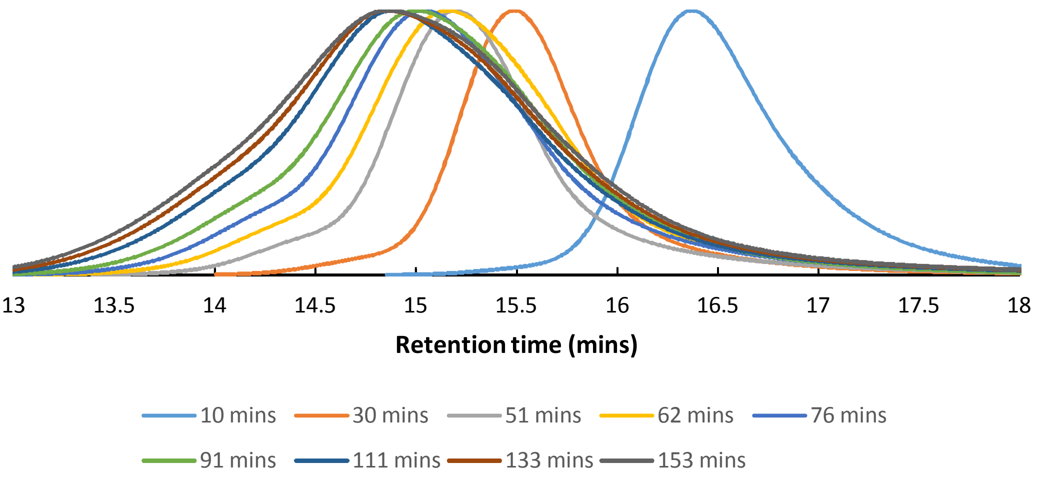

Figure S37. Overlay of SEC traces for the polymerisation of *rac*-LA mediated by **2**. Conditions: [LA]/[**2**] = 500, [LA] = 1 M, THF, 298 K.

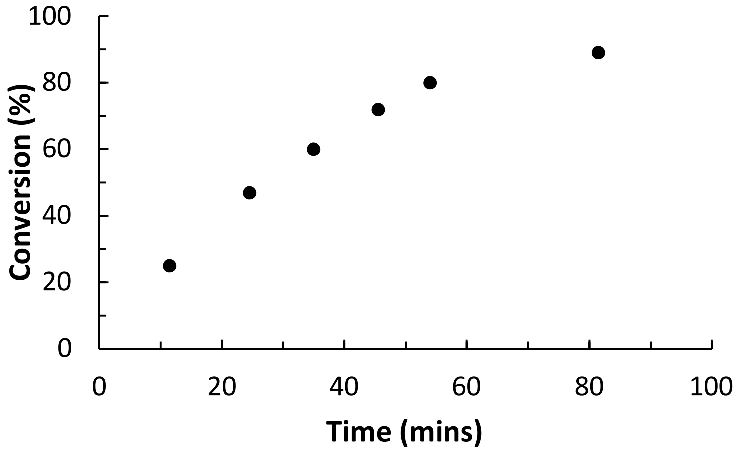

Figure S38. Conversion vs time plot for the polymerisation of *rac*-LA mediated by compound **2**. Conditions: [LA]/[**2**] = 350, [LA] = 1 M, THF, 298 K.

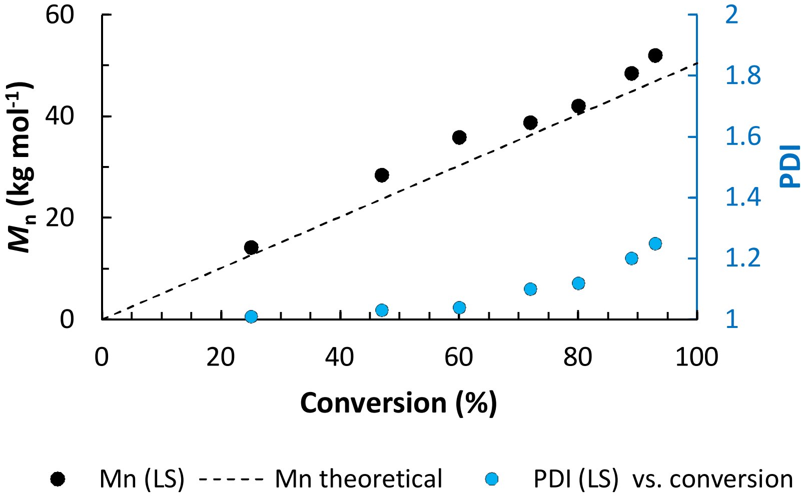

Figure S39. Plot of the molecular weight and polydispersity vs conversion for the polymerisation of *rac*-LA mediated by compound **2**. Conditions: [LA]/[**2**] = 350, [LA] = 1 M, THF, 298 K

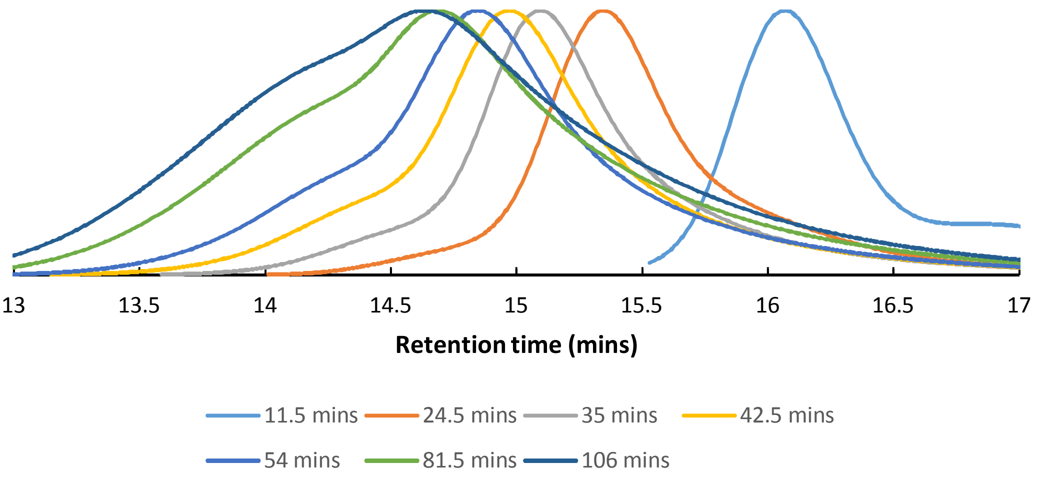

Figure S40. Overlay of SEC traces for the polymerisation of *rac*-LA mediated by **2**. Conditions: [LA]/[**2**] = 350, [LA] = 1 M, THF, 298 K.

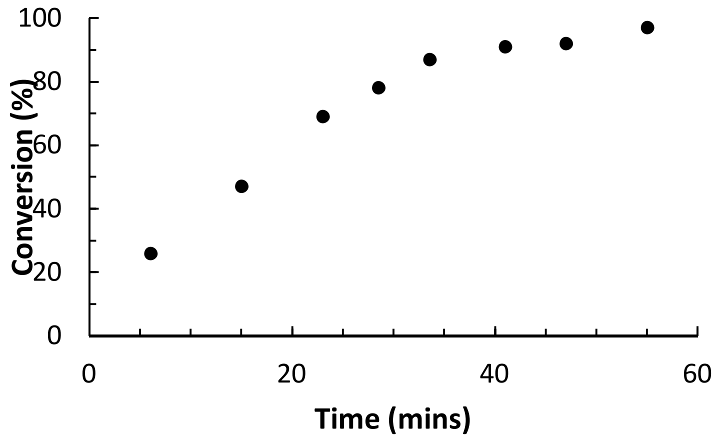

Figure S41. Conversion vs time plot for the polymerisation of *rac*-LA mediated by compound **2**. Conditions: [LA]/[**2**] = 250, [LA] = 1 M, THF, 298 K.

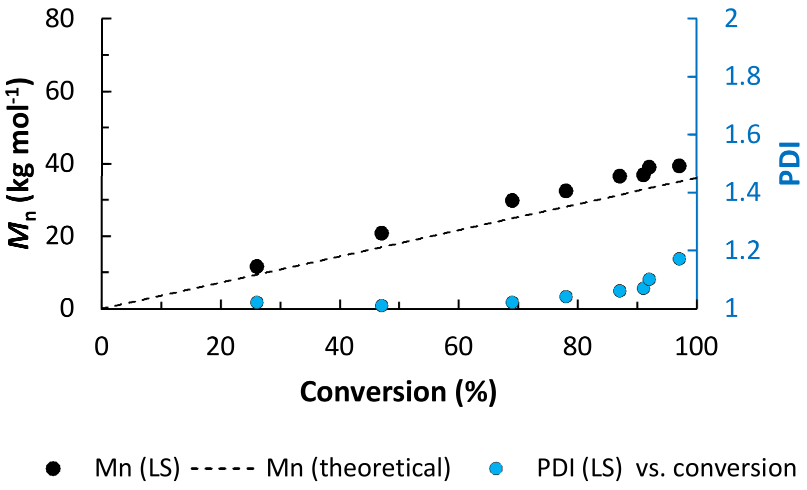

Figure S42. Plot of the molecular weight and polydispersity vs for the polymerisation of *rac*-LA mediated by compound **2**. Conditions: [LA]/[**2**] = 250, [LA] = 1 M, THF, 298 K.

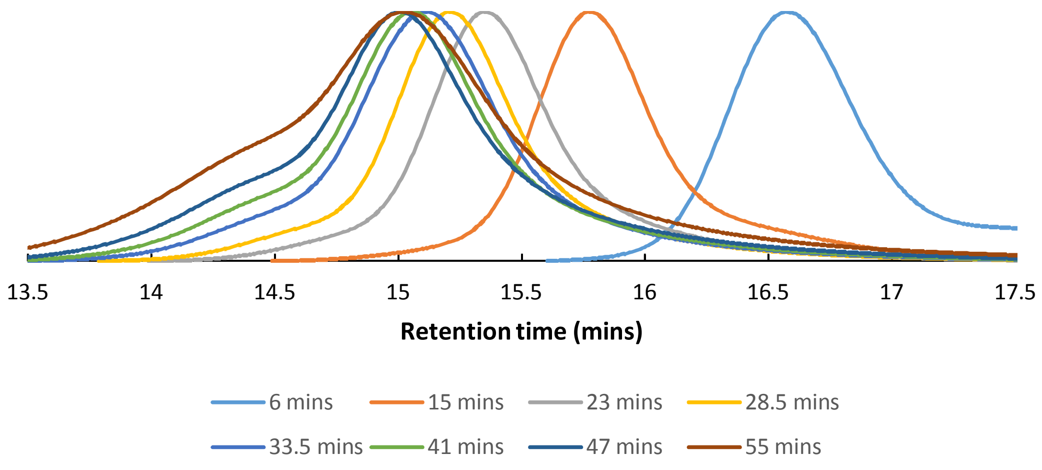

Figure S43. Overlay of SEC traces for the polymerisation of *rac*-LA mediated by **2**. Conditions: [LA]/[**2**] = 250, [LA] = 1 M, THF, 298 K.

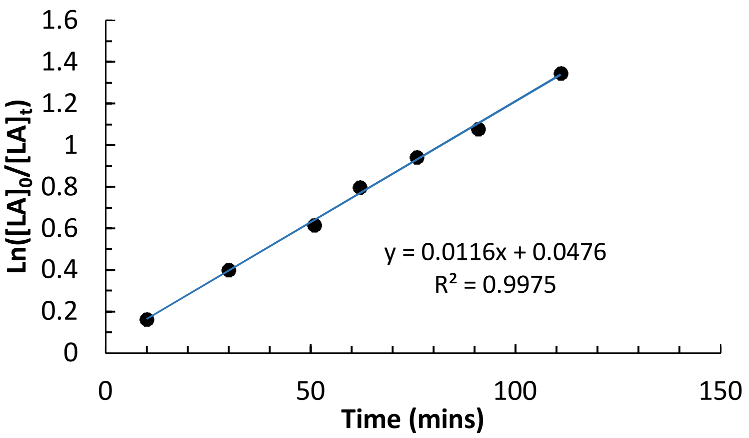

Figure S44. Semi-logarithmic first-order plot for the polymerisation of *rac*-LA mediated by compound **2**. Conditions: [LA]/[**2**] = 500, [LA] = 1 M, THF, 298 K.

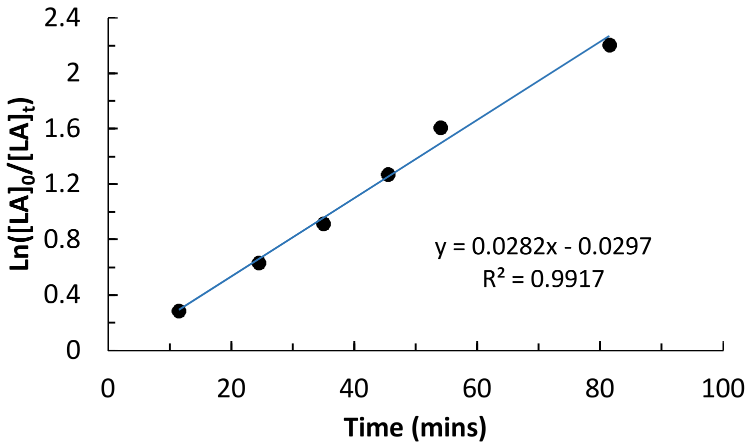

Figure S45. Semi-logarithmic first-order plot for the polymerisation of *rac*-LA mediated by compound **2**. Conditions: [LA]/[**2**] = 350, [LA] = 1 M, THF, 298 K.

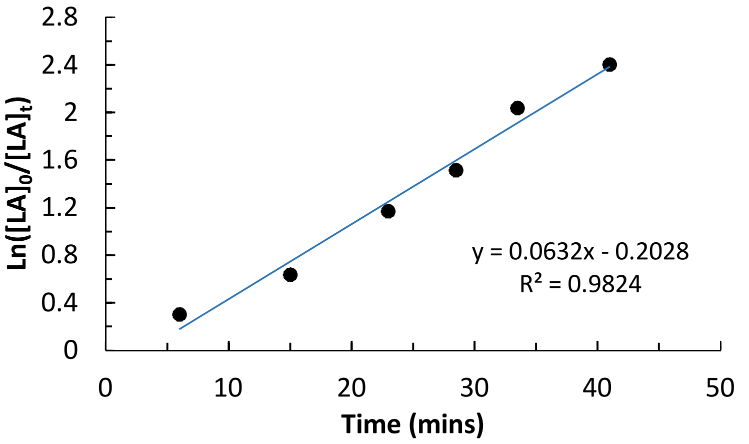

Figure S46. Semi-logarithmic first-order plot for the polymerisation of *rac*-LA mediated by compound **2**. Conditions: [LA]/[**2**] = 250, [LA] = 1 M, THF, 298 K.

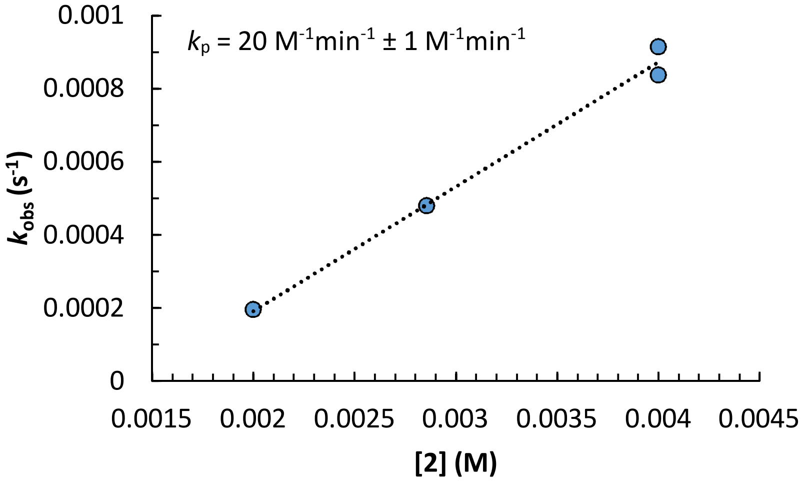

Figure S47. Plot of k_{obs} vs [**2**] for the polymerisation of *rac*-LA mediated by compound **2**. Conditions: [LA] = 1 M, THF, 298 K.

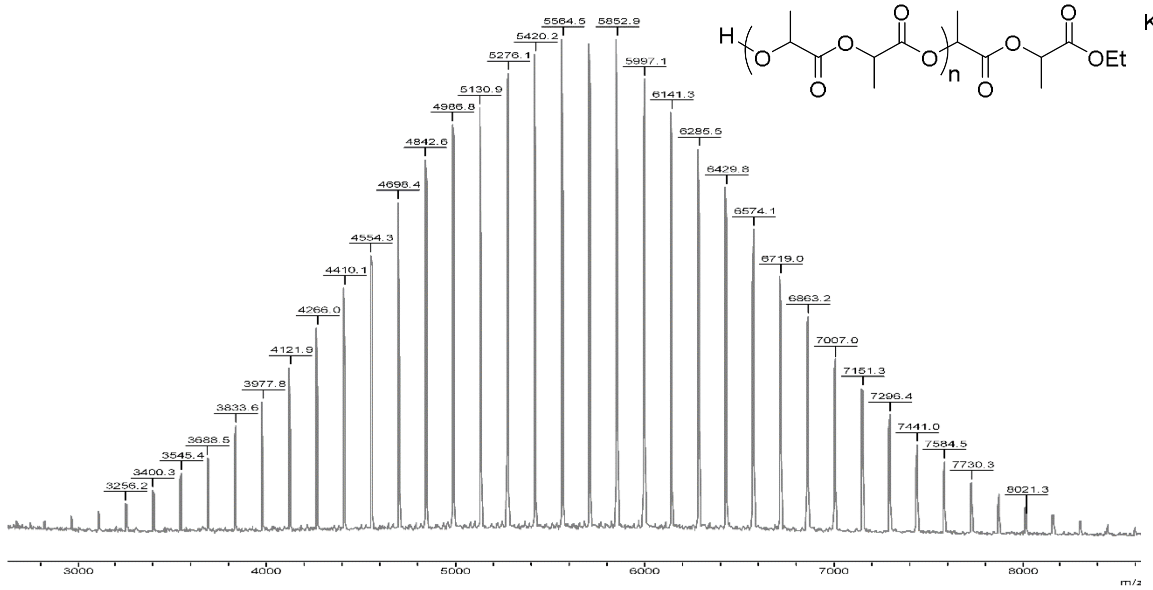

Figure S48. MALDI-ToF spectrum obtained from the polymerisation of *rac*-LA mediated by **1**. Conditions: [LA]/[**1**] = 500, [LA] = 0.75 M, THF, 244 K. Conv. = 8 %, time = 4 h, M_n = 6.5 kg mol^{-1} (SEC), PDI = 1.08 (SEC), M_n = 5.4 kg mol^{-1} (MALDI), PDI = 1.03 (MALDI), M_n ^{theo} = 5.8 kg mol^{-1}

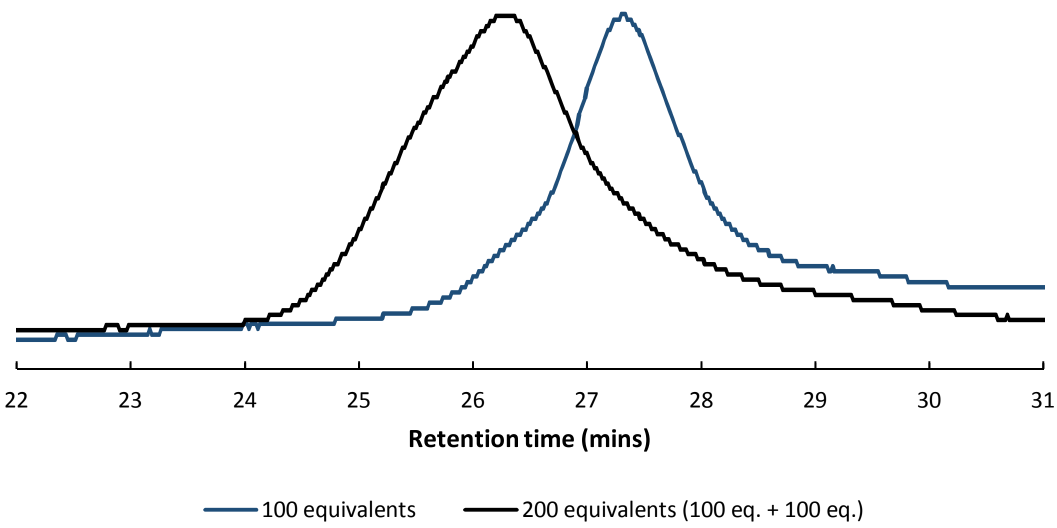

Figure S49. Overlay of SEC traces for the polymerisation of 100 eq. + 100 eq. of *rac*-LA with **1**. Conditions: [LA] = 0.5 M, THF, 298 K

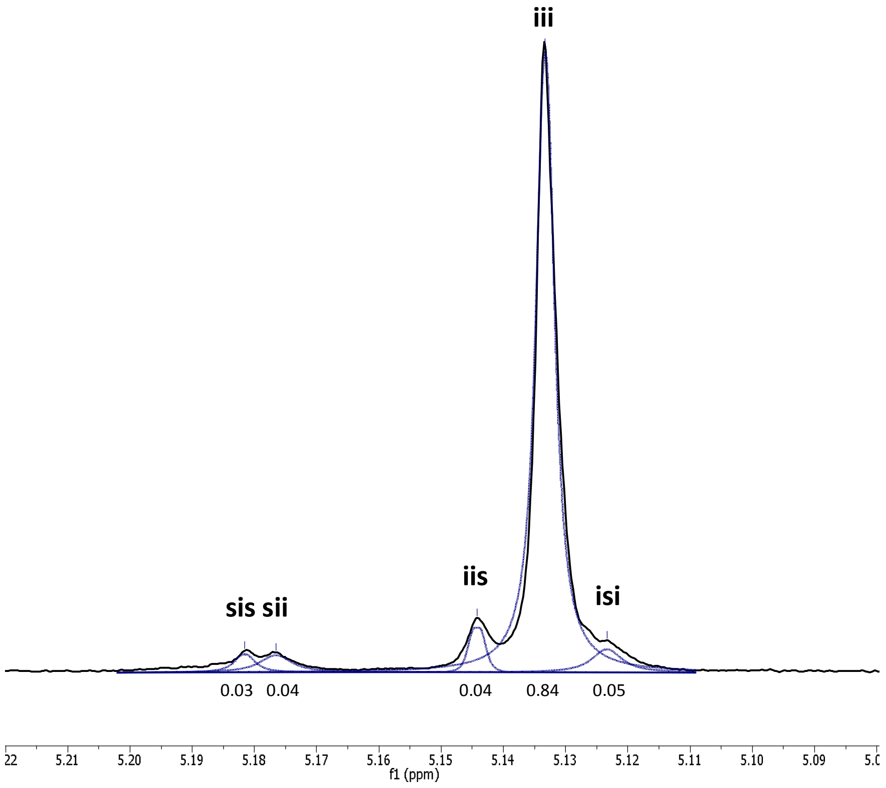

Figure S50. Deconvoluted homonuclear-decoupled ^{1}H NMR spectrum of the methine region of PLA obtained with **1** in $CDCl_3$; P_i values determined by evaluation of all tetrads. (Table 1, entry 1 in publication).

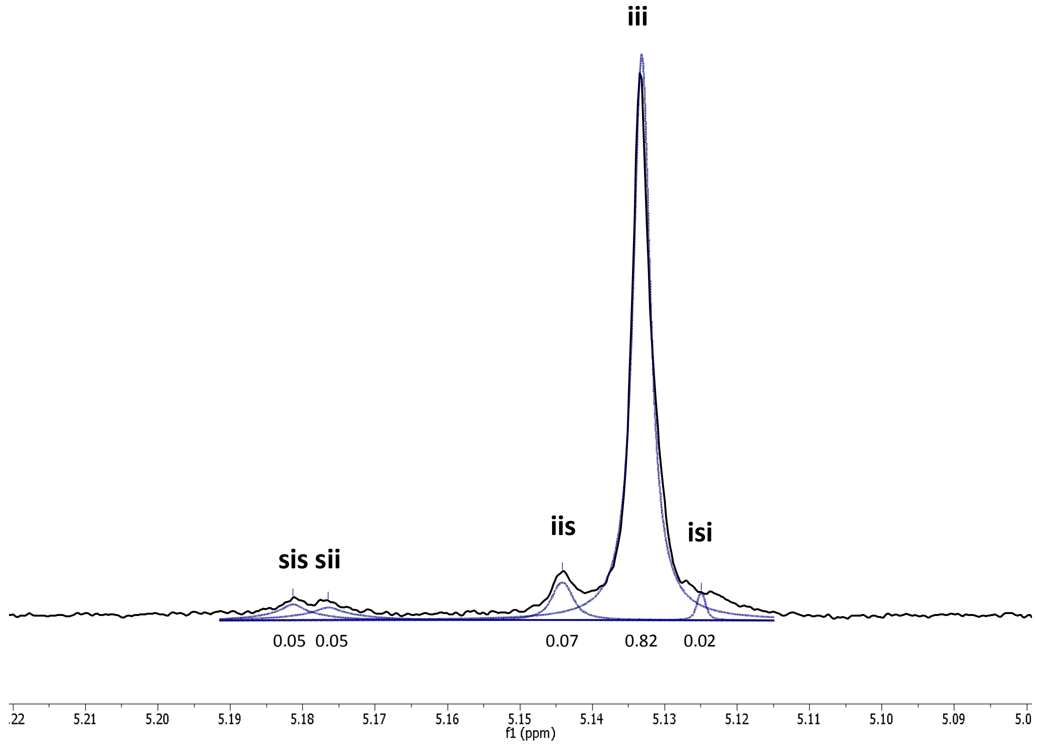

Figure S51. Deconvoluted homonuclear-decoupled ^{1}H NMR spectrum of the methine region of PLA obtained with **1** in $CDCl_3$; P_i values determined by evaluation of all tetrads. (Table 1, entry 2 in publication).

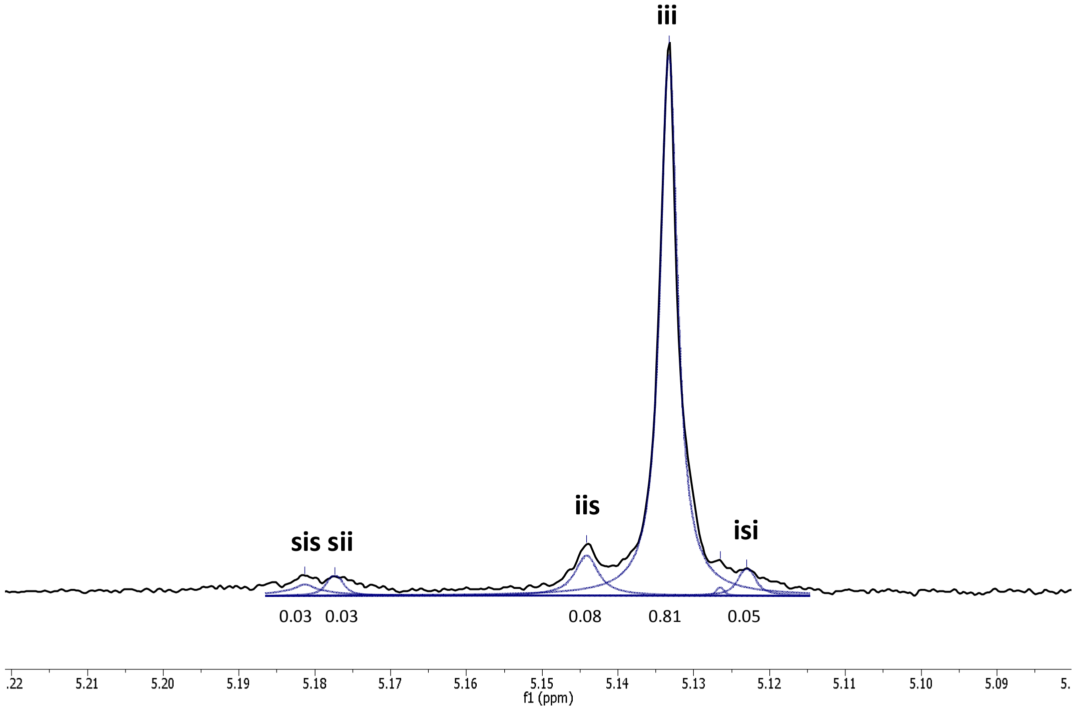

Figure S52. Deconvoluted homonuclear-decoupled ^{1}H NMR spectrum of the methine region of PLA obtained with **1** in $CDCl_3$; P_i values determined by evaluation of all tetrads. (Table 1, entry 3 in publication).

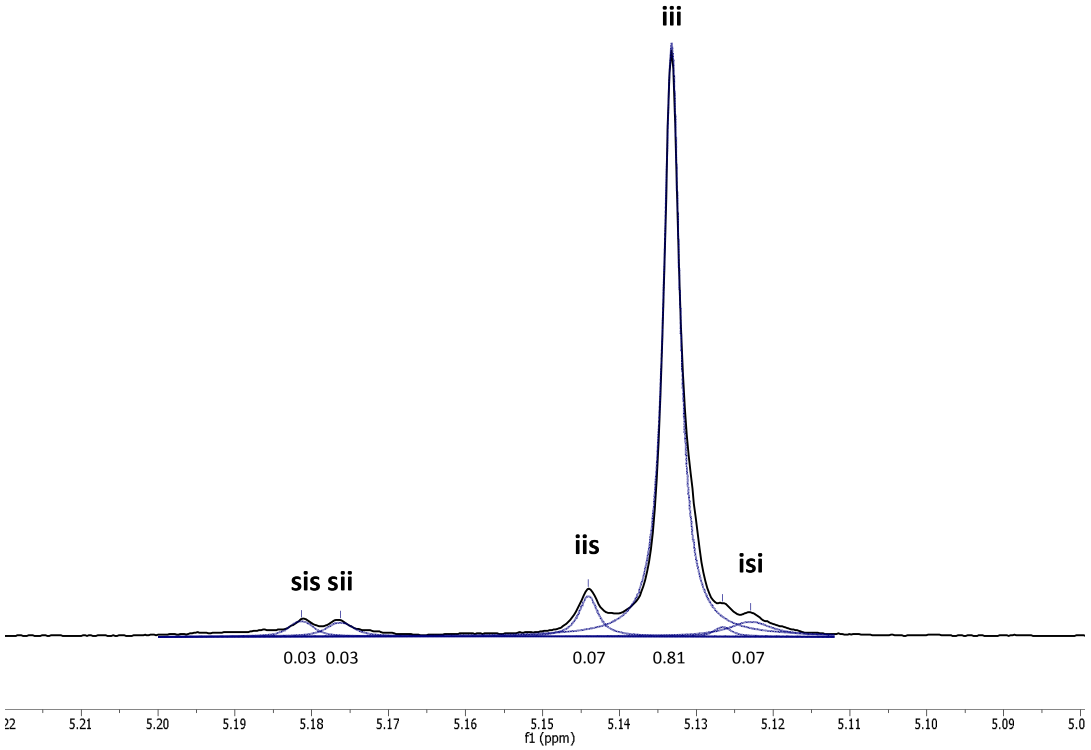

Figure S53. Deconvoluted homonuclear-decoupled ^1H NMR spectrum of the methine region of PLA obtained with **1** in $CDCl_3$; P_i values determined by evaluation of all tetrads. (Table 1, entry 4 in publication).

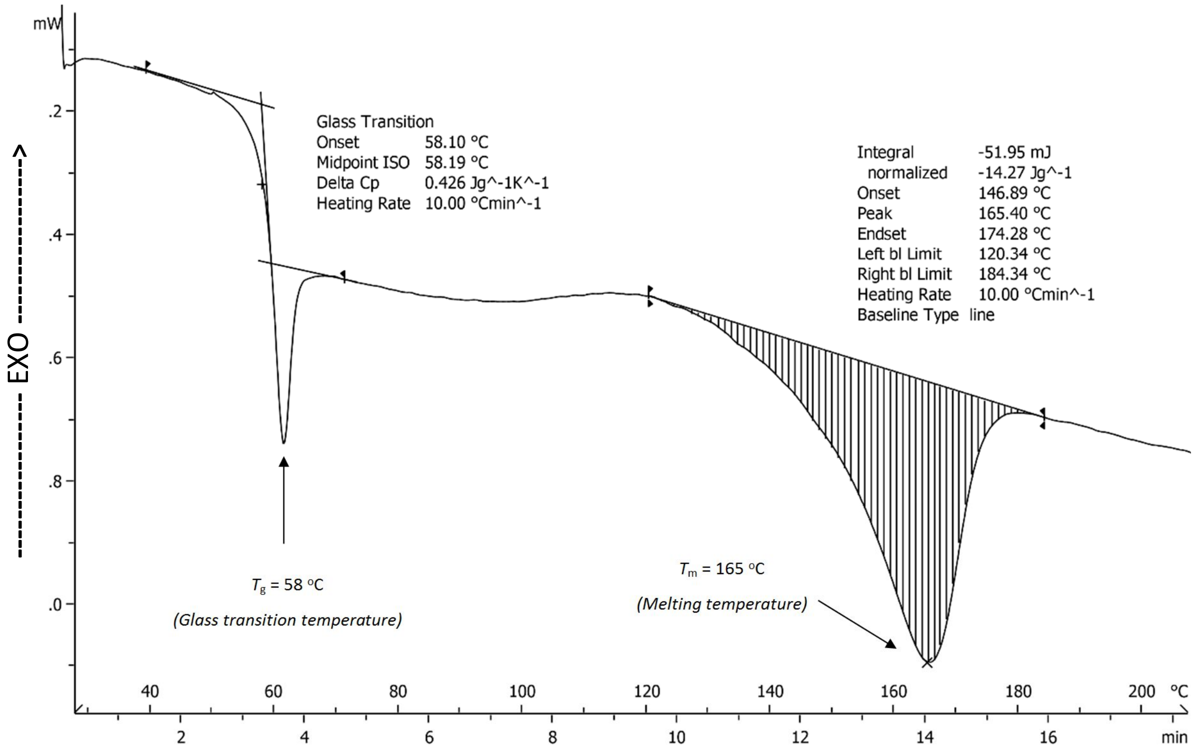

Figure S54. Heat flow vs temperature curve for a PLA sample obtained from **1**. (Table 1, entry 1 in publication).

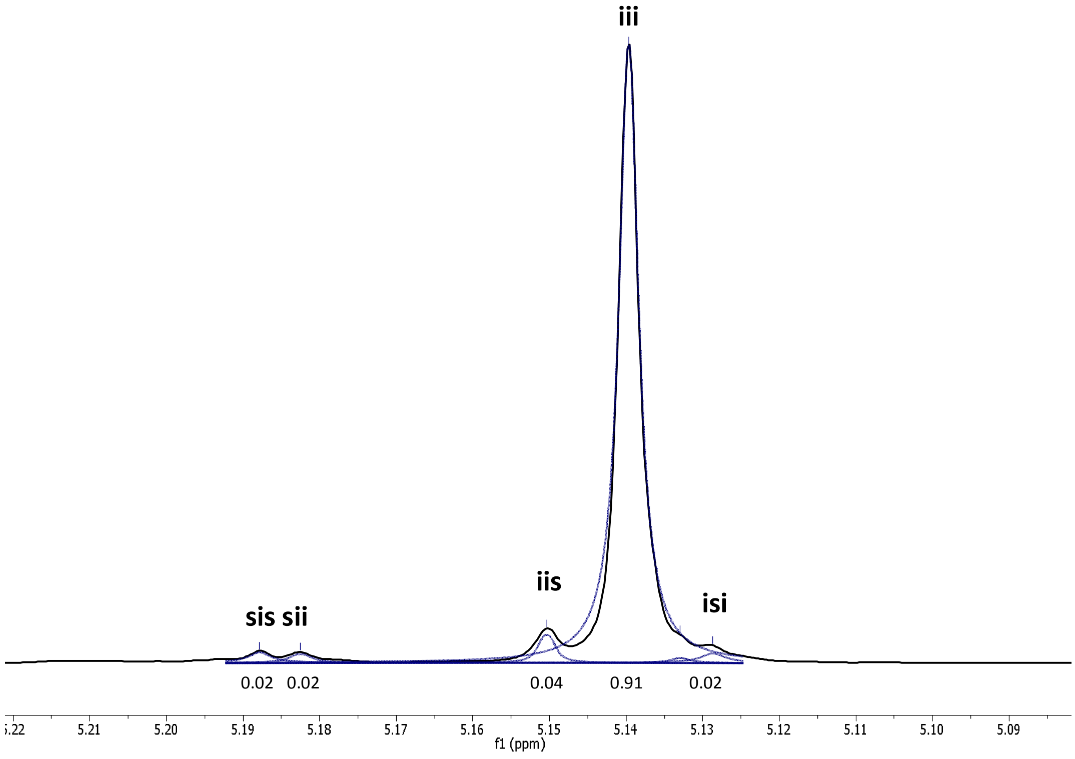

Figure S55. Deconvoluted homonuclear-decoupled ^{1}H NMR spectrum of the methine region of PLA obtained with **1** in $CDCl_3$; P_i values determined by evaluation of all tetrads. (Table 1, entry 7 in publication).

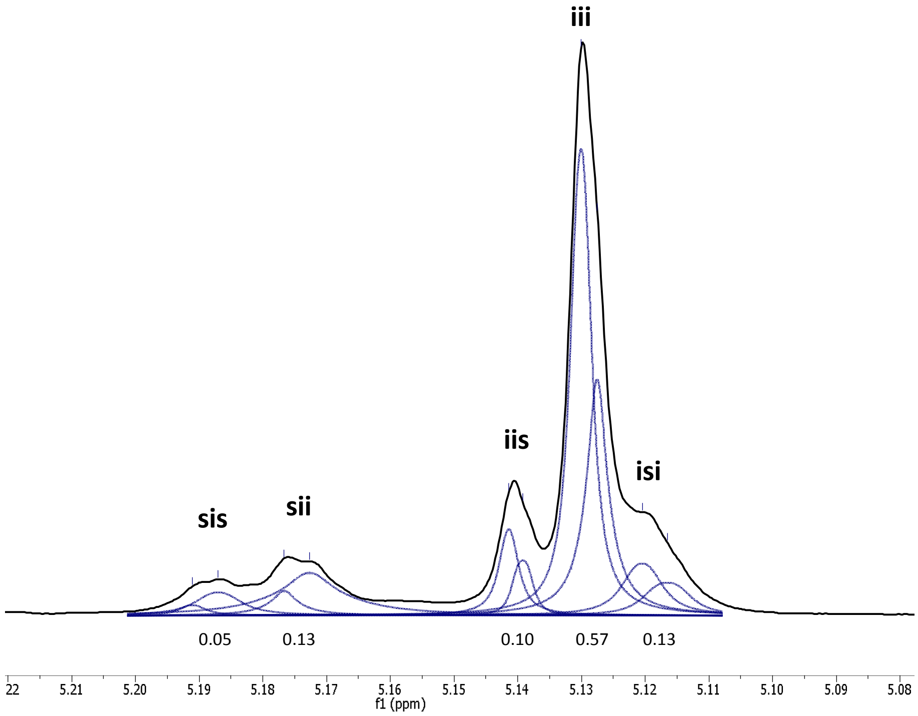

Figure S56. Deconvoluted homonuclear-decoupled ^{1}H NMR spectrum of the methine region of PLA obtained with **1** in $CDCl_3$; P_i values determined by evaluation of all tetrads. (Table 1, entry 8 in publication).

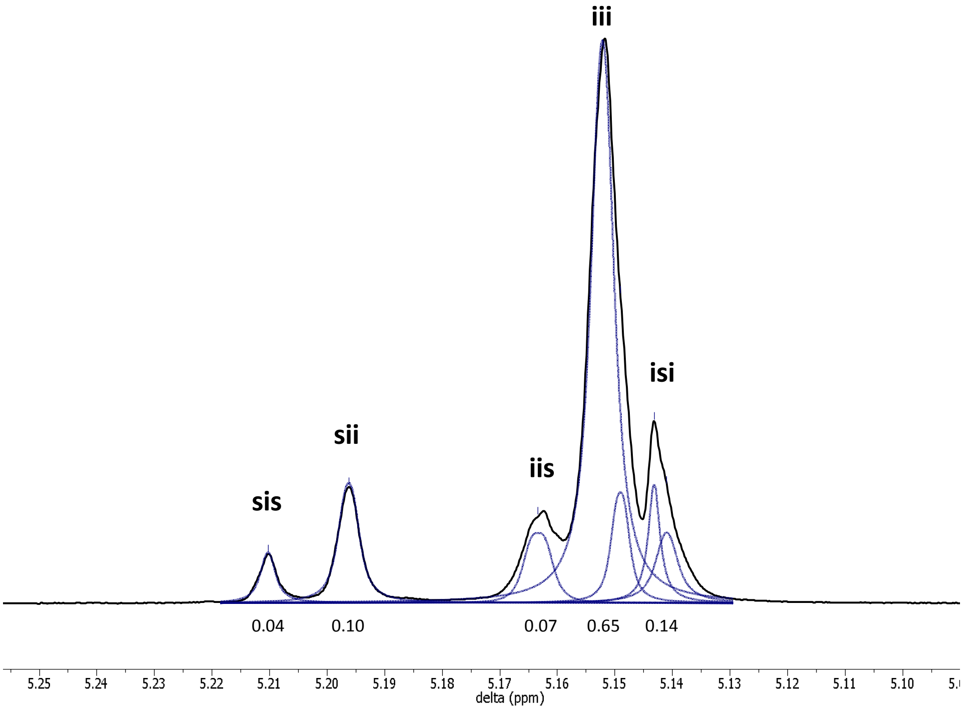

Figure S57. Deconvoluted homonuclear-decoupled ^{1}H NMR spectrum of the methine region of PLA obtained with **2** in $CDCl_3$; P_i values determined by evaluation of all tetrads. (Table 1, entry 9 in publication).

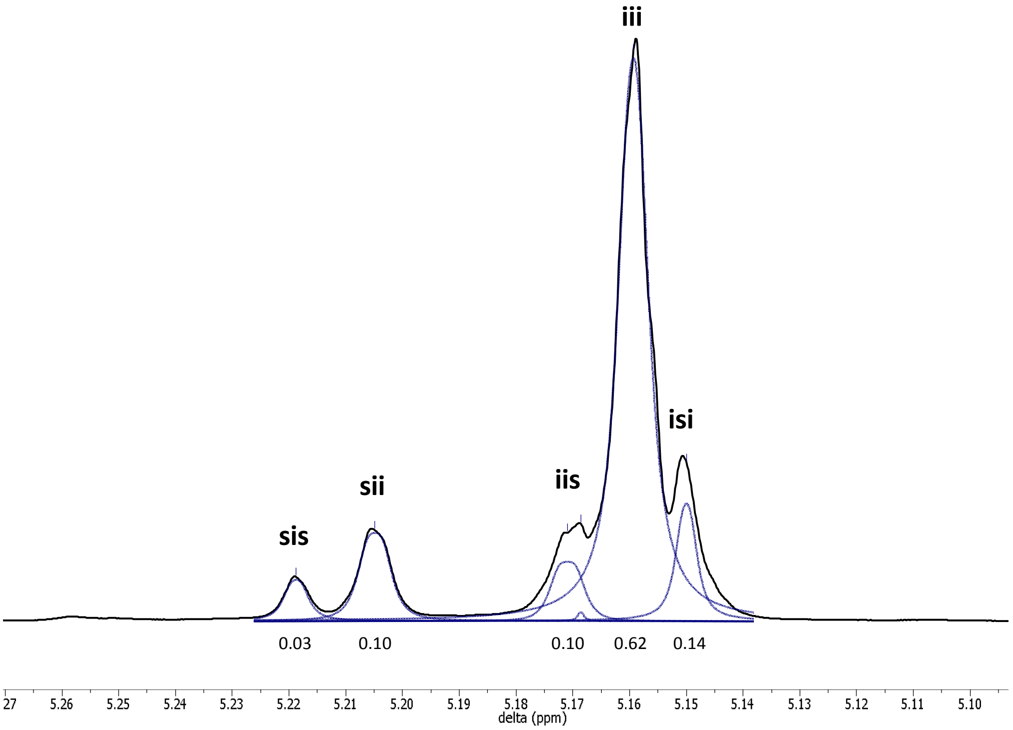

Figure S58. Deconvoluted homonuclear-decoupled ^{1}H NMR spectrum of the methine region of PLA obtained with **2** in $CDCl_3$; P_i values determined by evaluation of all tetrads. (Table 1, entry 10 in publication).

Bibliography

[1] C. Bakewell, T.-P.-A. Cao, N. Long, X. F. Le Goff, A. Auffrant, C. K. Williams, *J. Am. Chem. Soc.,* **2012**, *134*, 20577-20580.

[2] J. Baran, A. Duda, A. Kowalski, R. Szymanski, S. Penczek, *Macromol. Rapid Commun.,* **1997**, *333*, 325-333.

[3] J. R. Dorgan, J. Janzen, D. M. Knauss, S. B. Hait, B. R. Limoges, M. H. Hutchinson, *J. Polym. Sci., Part B: Polym. Phys.,* **2005**, *43*, 3100-3111.

[4] J. Coudane, C. Ustariz-Peyret, G. Schwach, M. Vert, *J. Polym. Sci., Part A: Polym. Chem.,* **1997**, *35*, 1651-1658.

[5] Bruker AXS, Madison WI, **2014**.

[6] G. M. Sheldrick, *Acta Crystallogr. Sect. C: Cryst. Struct. Commun.,* **2015**, *71*, 3-8.

[7] SHELXTL v5.1, Bruker AXS, Madison WI, **1998**.

[8] a) A. L. Spek, *J. Appl. Crystallogr.,* **2003**, *36*, 7-13; b) A. L. Spek, *Acta Crystallogr. Sect. D-Biol. Crystallogr.,* **2009**, *65*, 148-155; c) A. L. Spek, *Acta Crystallogr. Sect. C: Cryst. Struct. Commun.,* **2015**, *71*, 9-18.
